# Assessment of Coronary Artery Disease With Computed Tomography Angiography and Inflammatory and Immune Activation Biomarkers Among Adults With HIV Eligible for Primary Cardiovascular Prevention

**DOI:** 10.1001/jamanetworkopen.2021.14923

**Published:** 2021-06-29

**Authors:** Udo Hoffmann, Michael T. Lu, Borek Foldyna, Markella V. Zanni, Julia Karady, Jana Taron, Bingxue K. Zhai, Tricia Burdo, Kathleen V. Fitch, Emma M. Kileel, Kenneth Williams, Carl J. Fichtenbaum, Edgar T. Overton, Carlos Malvestutto, Judith Aberg, Judith Currier, Craig A. Sponseller, Kathleen Melbourne, Michelle Floris-Moore, Cornelius Van Dam, Michael C. Keefer, Susan L. Koletar, Pamela S. Douglas, Heather Ribaudo, Thomas Mayrhofer, Steven K. Grinspoon

**Affiliations:** 1 Massachusetts General Hospital, Boston; 2 MTA-SE Cardiovascular Imaging Research Group, Heart and Vascular Center, Semmelweis University, Budapest, Hungary; 3 University Hospital Freiburg, Freiburg, Germany; 4 Brigham and Women’s Hospital, Boston, Massachusetts; 5 Temple University, Philadelphia, Pennsylvania; 6 Boston College, Boston, Massachusetts; 7 University of Cincinnati, Cincinnati, Ohio; 8 University of Alabama, Birmingham; 9 Ohio State University, Columbus; 10 Icahn School of Medicine at Mount Sinai, New York, New York; 11 University of California at Los Angeles; 12 Kowa Pharmaceuticals America, Montgomery, Alabama; 13 Gilead Sciences, Foster City, California; 14 University of North Carolina, Chapel Hill; 15 Greensboro Clinical Research Site, Cone Health, Greensboro, North Carolina; 16 University of Rochester Adult HIV Therapeutic Strategies Network Clinical Research Site, Rochester, New York; 17 Duke Clinical Research Institute, Duke University School of Medicine, Durham, North Carolina; 18 Center for Biostatistics in AIDS Research, Harvard T.H. Chan School of Public Health, Boston, Massachusetts; 19 School of Business Studies, Stralsund University of Applied Sciences, Stralsund, Germany

## Abstract

**Importance:**

Cardiovascular disease (CVD) is increased among people with HIV (PWH), but little is known regarding the prevalence and extent of coronary artery disease (CAD) and associated biological factors in PWH with low to moderate traditional CVD risk.

**Objectives:**

To determine unique factors associated with CVD in PWH and to assess CAD by coronary computed tomography angiography (CTA) and critical pathways of arterial inflammation and immune activation.

**Design, Setting, and Participants:**

This cohort study among male and female PWH, aged 40 to 75 years, without known CVD, receiving stable antiretroviral therapy, and with low to moderate atherosclerotic cardiovascular disease (ASCVD) risk according to the 2013 American College of Cardiology/American Heart Association pooled cohort equation, was part of the Randomized Trial to Prevent Vascular Events in HIV (REPRIEVE), a large, ongoing primary prevention trial of statin therapy among PWH conducted at 31 US sites. Participants were enrolled from May 2015 to February 2018. Data analysis was conducted from May to December 2020.

**Exposure:**

HIV disease.

**Main Outcomes and Measures:**

The primary outcome was the prevalence and composition of CAD assessed by coronary CTA and, secondarily, the association of CAD with traditional risk indices and circulating biomarkers, including insulin, monocyte chemoattractant protein 1 (MCP-1), interleukin (IL) 6, soluble CD14 (sCD14), sCD163, lipoprotein-associated phospholipase A2 (LpPLA2), oxidized low-density lipoprotein (oxLDL), and high-sensitivity C-reactive protein (hsCRP).

**Results:**

The sample included 755 participants, with a mean (SD) age of 51 (6) years, 124 (16%) female participants, 267 (35%) Black or African American participants, 182 (24%) Latinx participants, a low median (interquartile range) ASCVD risk (4.5% [2.6%-6.8%]), and well-controlled viremia. Overall, plaque was seen in 368 participants (49%), including among 52 of 175 participants (30%) with atherosclerotic CVD (ASCVD) risk of less than 2.5%. Luminal obstruction of at least 50% was rare (25 [3%]), but vulnerable plaque and high Leaman score (ie, >5) were more frequently observed (172 of 755 [23%] and 118 of 743 [16%], respectively). Overall, 251 of 718 participants (35%) demonstrated coronary artery calcium score scores greater than 0. IL-6, LpPLA2, oxLDL, and MCP-1 levels were higher in those with plaque compared with those without (eg, median [IQR] IL-6 level, 1.71 [1.05-3.04] pg/mL vs 1.45 [0.96-2.60] pg/mL; *P* = .008). LpPLA2 and IL-6 levels were associated with plaque in adjusted modeling, independent of traditional risk indices and HIV parameters (eg, IL-6: adjusted odds ratio, 1.07; 95% CI, 1.02-1.12; *P* = .01).

**Conclusions and Relevance:**

In this study of a large primary prevention cohort of individuals with well-controlled HIV and low to moderate ASCVD risk, CAD, including noncalcified, nonobstructive, and vulnerable plaque, was highly prevalent. Participants with plaque demonstrated higher levels of immune activation and arterial inflammation, independent of traditional ASCVD risk and HIV parameters.

## Key Points

**Question:**

What is the extent of coronary artery disease among people with well-controlled HIV and low to moderate risk of atherosclerotic cardiovascular disease (ASCVD), and how is coronary artery disease associated with traditional risk, inflammatory, and immune activation indices?

**Findings:**

In this cohort study of 755 people with HIV, coronary plaque was highly prevalent. Critical stenosis was rare, but higher-risk plaque features, including vulnerable plaque and high Leaman scores, were seen in approximately one-fifth of participants; plaque indices were associated with ASCVD risk scores and, independently, indices of inflammation and immune activation.

**Meaning:**

These findings suggest that people with HIV at low to moderate risk of cardiovascular disease have a significant prevalence of coronary plaque associated with inflammation and immune activation markers.

## Teaser

This cohort study examines the prevalence of coronary artery disease (CAD) among adults with well-controlled HIV and low to moderate risk of atherosclerotic cardiovascular disease using computed tomography angiography and assessment of inflammation and immune activation biomarkers.

## Introduction

More than 38 000 000 people are infected with HIV worldwide. People with HIV (PWH) have significantly increased rates of cardiovascular disease (CVD),^1^ which may occur at a younger age in the context of lower traditional risk scores.^1^ The mechanisms of increased CVD rates in PWH are not entirely understood, but traditional risk factors are thought to explain only a portion of the risk increase.^2,3^ In this regard, PWH receiving effective antiretroviral therapy (ART) display residual immune activation^4^ and increased inflammatory indices,^5,6,7,8^ which have been associated with CVD in epidemiological studies^9,10^ and to coronary artery disease (CAD) in smaller studies.^11,12,13,14^ However, a comprehensive assessment of plaque and risk markers in a large, diverse, primary prevention cohort, comprised of men and women receiving ART with low to moderate cardiovascular risk scores has not been performed.

The Randomized Trial to Prevent Vascular Events (REPRIEVE) will test the hypothesis that statin therapy, with pleiotropic effects on inflammatory and cholesterol pathways, is a potent primary prevention strategy for major adverse cardiovascular events among PWH.^15^ The mechanistic substudy was designed to simultaneously assess plaque by coronary computed tomography angiography (CTA) and critical pathways of arterial inflammation and immune activation.^16^ In this baseline analysis, our primary objective was to assess the prevalence and composition of CAD, with a focus on the presence of plaque as our main CAD end point. Our secondary objective was to assess immune activation and inflammatory indices in association with plaque.

## Methods

Male and female PWH, aged 40 to 75 years, without known CVD and receiving stable ART, not receiving statin therapy, and with low to moderate cardiovascular risk were recruited based on estimated 10-year risk using the 2013 American College of Cardiology/American Heart Association (ACC/AHA) pooled cohort equation (PCE) and low-density lipoprotein cholesterol (LDL-C) levels (eTable 1 in Supplement 1).^15^ Participants with known active systemic infections and serious illness requiring systemic treatments other than HIV were excluded. The mechanistic substudy of REPRIEVE was performed at 31 US REPRIEVE sites, mostly from the AIDS Clinical Trial Group (ACTG) Network (eTable 2 in Supplement 1). Participants enrolling in REPRIEVE at those sites were offered enrollment into the mechanistic substudy. Exclusion criteria were glomerular filtration rate (eGFR) of less than 60 mL/min/1.73 m^2^, contrast allergy, arrhythmia precluding coronary CTA, contraindication to β-blockers for heart rate control during CTA, pregnancy, and body mass index (BMI; calculated as weight in kilograms divided by height in meters squared) 40 or greater. Enrollment occurred from May 2015 to February 2018. The study was approved by the Mass General Brigham Human Research Committee and by the local institutional review boards of each site. Informed consent was obtained in writing from each participant prior to participation in the study. This study followed the Strengthening the Reporting of Observational Studies in Epidemiology (STROBE) reporting guideline. Race and ethnicity were self-reported in accordance with guidelines in use by the ACTG.

### Coronary CTA

#### Acquisition

Details of site selection and qualification as well as quality-control measures for data acquisition have been previously described.^16^ Coronary CTA was performed on at least 64 slice CT scanners according to a standardized protocol consistent with the Society of Cardiovascular CT guidelines for clinical coronary CTA^17^ (eMethods in Supplement 1). Anonymized CTA images were transferred to the REPRIEVE CT core laboratory, which reviewed the images for completeness, quality, and radiation dose. Scans underwent an initial real-time read for critical stenosis, defined as any stenosis 70% or greater or left main stenosis 50% or greater. If identified, sites were notified of this clinically relevant finding as per a prespecified plan, with participants managed per site standard of care.

#### Assessment of the Presence, Extent, and Composition of CAD

Contrast-enhanced CTAs were reviewed for the presence and composition of atherosclerotic plaque and the degree of stenosis (none; mild, 1%-49%; moderate, 50%-69%; severe: ≥50% left main or ≥70% in any other coronary segment) using the standard 18-segment coronary model.^18^ We assessed the presence of vulnerable plaque features, defined based on any 1 of 3 features: positive remodeling (remodeling index, >1.1), CT attenuation of less than 30 Hounsfield units, and napkin-ring sign (low central attenuation with ring-like peripheral high attenuation).^19^ We calculated the simple segment involvement score (SIS), reflecting the total number of segments with coronary plaque per patient.^20^ We also reported the more comprehensive CT Leaman score, which accounts for the degree of stenosis, coronary dominance, plaque location, and composition.^21^ The coronary artery calcium (CAC) score was quantified on noncontrast CT using a modified Agatston method.^22^ All CT data sets were randomly assigned to 1 of 3 experienced CT core laboratory readers (B.F., J.K., and J.T.). To ensure consistency, readers completed a standardized certification process, including reading 20 training data sets. Interobserver variability was established in an additional 20 REPRIEVE CTAs analyzed by all readers, with good agreement for coronary plaque presence (Cohen κ = 0.89). All analyses were performed on a dedicated workstation (Aquarius iNtuition, TeraRecon).^23^

### Clinical and Biomarker Data

Screening CD4 and viral load were obtained from clinical care. Prespecified inflammatory and immune activation biomarkers representing potentially statin-modifiable pathways, including monocyte chemoattractant protein-1 (MCP-1), interleukin (IL) 6, soluble CD14 (sCD14), sCD163, lipoprotein-associated phospholipase A2 (LpPLA2), and oxidized low-density lipoprotein (oxLDL) were drawn fasting and measured centrally in duplicate from plasma using enzyme-linked immunosorbent assay kits at Temple University (Philadelphia, Pennsylvania). Insulin and high-sensitivity C-reactive protein (hsCRP) were performed from serum at Quest Diagnostics. Assay limits and variability are shown in eTable 3 in Supplement 1.

### Statistical Analysis

Continuous variables are presented as means with SDs or medians with interquartile ranges (IQRs). Categorical variables are presented as absolute and relative frequencies. Comparisons between groups were performed with the use of a 2-sample *t* test or Wilcoxon rank sum test for continuous variables and Fisher exact test for categorical variables. Trends across ASCVD risk groups were tested using an extension of the Wilcoxon rank-sum test developed by Cuzick.^24^ Adjusted logistic regression models assessed the association of biomarkers with CAD, represented by the presence of plaque as an overall index of CAD. Supplemental analyses were performed similarly assessing these associations to (1) CAC, (2) vulnerable plaque, and (3) Leaman score greater than 5 among all participants. Assumptions of linearity between transformed biomarkers and the log-odds were assessed by the Box-Tidwell test. Biomarkers were log2 transformed and then divided by 0.32 to give effects per 25% increase of biomarker value. Effects are also given per SD of the log-transformed values. hsCRP was used as a stratified variable corresponding to low, average, and high risk categories in comparison with plaque indices and as a continuous variable in regression modeling (eMethods in Supplement 1) for CAD. We further assessed the association of biomarkers (exposure) to plaque (outcome), adjusting for key demographic and clinical covariates. The purpose of the modeling was to adjust for potential confounding rather than performing mediation analysis. First, biomarkers were identified that were significantly different between those with and without plaque. These associations were further interrogated in 3 adjusted models, as follows: model 1 included all biomarkers with statistically significant associations in unadjusted analyses plus ASCVD risk score and HIV-related parameters (ie, current CD4, nadir CD4, duration of ART as categorical variables); model 2, all variables from model 1 plus age, sex, and race; and model 3, all biomarkers with statistically significant associations in unadjusted analyses plus age, sex, race, LDL-C level, hypertension, current smoking, and HIV-related parameters. Sensitivity analyses were conducted including all biomarkers in each model and testing for interaction terms between each biomarker and age, sex, and race. Inference was guided with a 2-sided 5% false-positive error rate without adjustment for multiple comparisons (ie, not a familywise error rate) and clinically meaningful effect sizes. Statistical analyses were performed using Stata version 16.1 (StataCorp). The sample size of 800 was determined for the assessment of pitavastatin calcium effects on plaque.

## Results

### Study Population

Participants were included in the current analysis based on the availability of the baseline CTA scan data. Of 805 enrolled participants, 780 (97%) completed a baseline coronary CTA. Of those, 755 (97%) had a diagnostic coronary CTA that permitted assessment of presence, extent, and composition of coronary atherosclerosis (eFigure 1 in Supplement 1), which defined the primary analytic cohort of this study. Plasma biomarkers were available for 746 (99%) for insulin and hsCRP and 747 (99%) for the other analytes.

### Demographic and Clinical Parameters

Baseline demographic characteristics are shown in Table 1. The cohort had a mean (SD) age of 51 (6) years and included 124 (16%) female participants. Approximately half of the participants were White (406 [54%]), with 267 Black or African American participants (35%), 10 Asian participants (1%), and 72 participants (10%) identifying as another race. Approximately one-quarter of participants (182 [24%]) were Latinx. The average participant had a low estimated ASCVD risk (median [IQR] PCE risk score, 4.5% [2.6%-6.8%]), with 150 participants (20%) having a PCE risk score of 7.5% or greater. The mean (SD) LDL-C level was 108 (30) mg/dL (to convert to millimoles per liter, multiply by 0.0259), and the population had a low prevalence of diabetes (3 participants [0.4%]) based on the inclusion criteria of REPRIEVE to enroll a primary prevention cohort. All participants were receiving ART, and nearly 60% (436 [58%]) had been receiving ART for more than 10 years with good virologic control (Table 1). The mechanistic substudy population was generally representative of participants enrolled in REPRIEVE in the US, with similar distributions of age, ART, CD4 levels, HIV RNA levels, and ASCVD risk. The substudy did include a lower percentage of female (124 [16%] vs 856 of 3788 [23%]) and a higher percentage of White (406 [54%] vs 1868 [49%]) participants compared with the overall US REPRIEVE population, with relatively fewer Black or African American and more Latinx participants.

**Table 1.** Demographic and Cardiovascular Characteristics by Enrollment Status

Characteristic	Participants, No. (%)^a^			
	Total US REPRIEVE population (n = 3788)	Substudy participants with CT results (n = 755)	Substudy site participants not enrolled or without CT results (n = 1293)	Participants at other US sites (n = 1740)
**Demographic and behavioral characteristics**				
Age, mean (SD), y	51 (6)	51 (6)	51 (6)	51 (6)
Women	856 (23)	124 (16)	343 (27)	389 (22)
Men	2932 (77)	631 (84)	950 (73)	1351 (78)
Gender identity				
Cisgender	3441 (91)	722 (96)	1207 (93)	1512 (87)
Transgender spectrum	83 (2)	15 (2)	29 (2)	39 (2)
Not reported	264 (7)	18 (2)	57 (4)	189 (11)
Race				
White	1868 (49)	406 (54)	594 (46)	868 (50)
Black or African American	1665 (44)	267 (35)	592 (46)	806 (46)
Asian	37 (1)	10 (1)	13 (1)	14 (1)
Other^b^	218 (6)	72 (10)	94 (7)	52 (3)
Ethnicity^c^				
Hispanic or Latino	692 (18)	182 (24)	261 (20)	249 (14)
Not Hispanic or Latino	3062 (81)	563 (75)	1027 (79)	1472 (85)
Unknown	34 (1)	10 (1)	5 (1)	19 (1)
Smoking status				
Current	1136 (30)	181 (24)	394 (31)	561 (32)
Former	1121 (30)	235 (31)	398 (31)	488 (28)
Never	1524 (40)	337 (45)	497 (39)	690 (40)
Substance use				
Current	105 (3)	16 (2)	45 (3)	44 (3)
Former	1893 (50)	367 (49)	618 (48)	908 (52)
Never	1782 (47)	369 (49)	626 (49)	787 (45)
**Cardiovascular and metabolic characteristics**				
ASCVD risk score,%				
Median (IQR)	5.0 (2.8-7.3)	4.5 (2.6-6.8)	5.1 (2.8-7.3)	5.1 (2.8-7.6)
0 to <2.5	815 (22)	175 (23)	267 (21)	373 (21)
2.5 to <5	1066 (28)	247 (33)	352 (27)	467 (27)
5 to 10	1605 (42)	286 (38)	584 (45)	735 (42)
>10	302 (8)	47 (6)	90 (7)	165 (9)
BMI, mean (SD)	28.0 (6.0)	27.3 (4.4)	28.4 (6.6)	28.0 (6.1)
Prior statin use	316 (8)	59 (8)	116 (9)	141 (8)
Hypertension^d^	1451 (38)	238 (32)	513 (40)	700 (40)
Diabetes	49 (1)	3 (0.4)	17 (1)	29 (2)
**HIV-related health history**				
Time since HIV diagnosis, median (IQR), y	15 (9-22)	15 (9-22)	16 (9-22)	15 (8-22)
Nadir CD4 count, cells/mm^3^				
<50	818 (22)	163 (22)	304 (24)	351 (20)
50-199	987 (26)	218 (29)	342 (26)	427 (25)
200-349	913 (24)	202 (27)	318 (25)	393 (23)
≥350	879 (23)	148 (20)	270 (21)	461 (26)
Unknown	191 (5)	24 (3)	59 (5)	108 (6)
Total ART use duration, y				
<5	649 (17)	120 (16)	207 (16)	322 (19)
5-10	1033 (27)	199 (26)	339 (26)	495 (28)
>10	2105 (56)	436 (58)	746 (58)	923 (53)
Unknown	1 (1)	0	1 (1)	0
Thymidine exposure	1395 (37)	289 (38)	520 (40)	586 (34)
Abacavir exposure	1244 (33)	253 (34)	405 (31)	586 (34)
TDF exposure	3399 (90)	694 (92)	1188 (92)	1517 (87)
Protease inhibitor exposure	2272 (60)	464 (62)	795 (62)	1013 (58)
**HIV-related health at REPRIEVE entry**				
CD4 count, cells/mm^3^				
<350	552 (15)	112 (15)	163 (13)	277 (16)
350-499	698 (18)	148 (20)	228 (18)	322 (19)
≥500	2538 (67)	495 (66)	902 (70)	1141 (66)
HIV-1 RNA, copies/mL				
<LLQ	3095 (85)	658 (88)	1066 (88)	1371 (81)
LLQ to <400	465 (13)	71 (10)	131 (11)	263 (16)
>400	83 (2)	16 (2)	15 (1)	52 (3)
ART regimen				
NRTI with INSTI	1716 (45)	335 (44)	603 (47)	778 (45)
NRTI with NNRTI	918 (24)	196 (26)	312 (24)	410 (24)
NRTI with PI	642 (17)	127 (17)	216 (17)	299 (17)
NRTI sparing	133 (4)	22 (3)	41 (3)	70 (4)
Other NRTI containing	379 (10)	75 (10)	121 (9)	183 (11)
Entry NRTI				
TDF	1854 (49)	379 (50)	649 (50)	826 (47)
TAF	1012 (27)	211 (28)	341 (26)	460 (26)
Abacavir	725 (19)	129 (17)	234 (18)	362 (21)
No NRTI	156 (4)	27 (4)	53 (4)	76 (4)
Other	41 (1)	9 (1)	16 (1)	16 (1)

Abbreviations: ART, antiretroviral therapy; ASCVD, atherosclerotic cardiovascular disease; BMI, body mass index (calculated as weight in kilograms divided by height in meters squared); CT, computed tomography; INSTI, integrase strand inhibitor; LLQ, lower limit of quantification; NNRTI, non–nucleoside reverse transcriptase inhibitor; NRTI, nucleoside reverse transcriptase inhibitor; PI, protease inhibitor; RNA, ribonucleic acid; TAF, tenofovir alafenamide; TDF, tenofovir disoproxil fumarate.

^a^ All statistics were calculated out of participants with data collected. Missing data (for substudy participants only): smoking status (2); substance use (3); HIV-1 RNA (10).

^b^ Other race includes participants self-identifying as Native or Indigenous to the enrollment region; more than 1 race (with no single race noted as predominant); or of unknown race.

^c^ Ethnicity presented per National Institutes of Health definition.

^d^ Hypertension defined as current diagnosis of hypertension, currently receiving an antihypertensive, or blood pressure greater than 140/90 mm Hg.

### CAD Characteristics

Nearly half the participants (368 [49%]) had evidence of plaque on coronary CTA (Table 2). Among 356 participants with quantifiable stenosis, almost all had nonobstructive CAD (331 [93%]). A CAC score greater than 0 was detected in 251 of 718 participants (35%). In contrast, the presence of advanced CAD, defined as CAC score of greater than 400 or luminal obstruction of 50% or greater was low (CAC >400, 13 of 718 participants [2%]; luminal obstruction ≥50%, 25 of 743 participants [3%]). Nearly one-quarter of participants (172 of 755 [23%]) had vulnerable plaques. In addition, noncalcified plaques were seen in 302 participants (40%) (Table 2). The mean (SD) Leaman score was 2.1 (2.8), with 118 of 743 (16%) having a Leaman score greater than 5. The mean (SD) SIS was 1.0 (1.4), with 101 of 755 participants (13%) presenting with at least 3 coronary plaques.

**Table 2.** Comparison of Coronary Artery Disease Indices by ASCVD Risk Score Categories

Variable	Participants, No./total No. (%)					*P* value
	All participants (N = 755)	ASCVD risk, 0% to <2.5% (n = 175)	ASCVD risk 2.5% to <5% (n = 247)	ASCVD risk 5% to <7.5% (n = 183)	ASCVD risk ≥7.5% (n = 150)	
Participants with any plaque	368 (48.7)	52 (29.7)	117 (47.4)	103 (56.3)	96 (64.0)	<.001
Plaque score categories						
0 Segments with plaque	387 (51.3)	123 (70.3)	130 (52.6)	80 (43.7)	54 (36.0)	<.001
1-2 Segments with plaque	267 (35.4)	44 (25.1)	85 (34.4)	75 (41.0)	63 (42.0)	
≥3 Segments with plaque	101 (13.4)	8 (4.6)	32 (13.0)	28 (15.3)	33 (22.0)	
Noncalcified plaque score categories						
0 Segments with noncalcified plaque	453 (60.0)	135 (77.1)	147 (59.5)	96 (52.5)	75 (50.0)	<.001
1-2 Segments with noncalcified plaque	244 (32.3)	35 (20.0)	81 (32.8)	71 (38.8)	57 (38.0)	
≥3 Segments with noncalcified plaque	58 (7.7)	5 (2.9)	19 (7.7)	16 (8.7)	18 (12.0)	
Vulnerable plaque features						
Participants with vulnerable plaque	172 (22.8)	22 (12.6)	52 (21.1)	44 (24.0)	54 (36.0)	<.001
Positive remodeling	166/172 (96.5)	21/22 (95.5)	51/52 (98.1)	42/44 (95.5)	52/54 (96.3)	NA
Low-attenuation plaque	45/172 (26.2)	4/22 (18.2)	11/52 (21.2)	11/44 (25.0)	19/54 (35.2)	NA
Napkin ring sign	23/172 (13.4)	2/22 (9.1)	5/52 (9.6)	5/44 (11.4)	11/54 (20.4)	NA
Positive remodeling and low-attenuation plaque	40/172 (23.4)	3/22 (13.6)	10/52 (19.2)	9/44 (20.5)	18/54 (33.3)	.04
Leaman score						
Mean (SD)	2.1 (2.8)	1.1 (2.2)	2.0 (2.8)	2.4 (2.9)	2.9 (3.0),	<.001
Median (IQR)	0.0 (0.0-3.2)	0.0 (0.0-2.2)	0.0 (0.0-3.2)	1.3 (0.0-3.8)	2.8 (0.0-4.6)	
0	387/743 (52.1)	123/174 (70.7)	130/244 (53.3)	80/180 (44.4)	54/145 (37.2)	<.001
>0-5	238/743 (32.0)	37/174 (21.3)	80/244 (32.8)	64/180 (35.6)	57/145 (39.3)	
>5	118/743 (15.9)	14/174 (8.1)	34/244 (13.9)	36/180 (20.0)	34/145 (23.5)	
Segment involvement score						
Mean (SD)	1.0 (1.4)	0.5 (1.0)	0.9 (1.5)	1.1 (1.5)	1.4 (1.6)	<.001
Median (IQR)	0.0 (0.0-1.0)	0.0 (0.0-1.0)	0.0 (0.0-1.0)	1.0 (0.0-2.0)	1.0 (0.0-2.0)	
Stenosis						
Participants with CAD and stenosis >0%	356/743 (47.9)	51/174 (29.3)	114/244 (46.7)	100/180 (55.6)	91/145 (62.8)	<.001
CAD categories in participants with CAD						
Mild CAD, stenosis 1%-49%	331/356 (93.0)	51/51 (100.0)	107/114 (93.9)	91/100 (91.0)	82/91 (90.1)	.03
Moderate CAD, stenosis 50%-69%	16/356 (4.5)	0/51	5/114 (4.4)	5/100 (5.0)	6/91 (6.6)	
Severe CAD, stenosis ≥70% or ≥50% left main	9/356 (2.5)	0/51	2/114 (1.8)	4/100 (4.0)	3/91 (3.3)	
CAD stenosis ≥50%	25/743 (3.4)	0/174	7/244 (2.9)	9/180 (5.0)	9/145 (6.2)	.001
CAC score						
>0	251/718 (35.0)	37/169 (21.9)	76/237 (32.1)	69/169 (40.8)	69/143 (48.3)	<.001
1-100	177/251 (70.5)	30/37 (81.1)	59/76 (77.6)	47/69 (68.1)	41/69 (59.4)	.004
101-400	61/251 (24.3)	7/37 (18.9)	14/76 (18.4)	18/69 (26.1)	22/69 (31.9)	
>400	13/251 (5.2)	0/37	3/76 (4.0)	4/69 (5.8)	6/69 (8.7)	

Abbreviations: ASCVD, atherosclerotic cardiovascular disease; CAC, coronary artery calcium; CAD, coronary artery disease; NA, not applicable.

### CAD and ASCVD Risk Category

Presence of plaque (Figure) as well as the degree of stenosis, extent of CAC, vulnerable plaque features, and composition and distribution, as summarized in the Leaman score, were higher with increasing ASCVD risk categories (Table 2; eFigure 2 in Supplement 1). Importantly, coronary plaques were found in 52 of 175 participants (30%) with a very low ASCVD risk (<2.5%) and 22 of 175 participants (13%) had vulnerable plaque features. In comparison, among the group with ASCVD risk less than 7.5%, 272 of 605 (45%) demonstrated plaque, and 118 of these 605 (20%) had vulnerable plaque.

**Figure.**
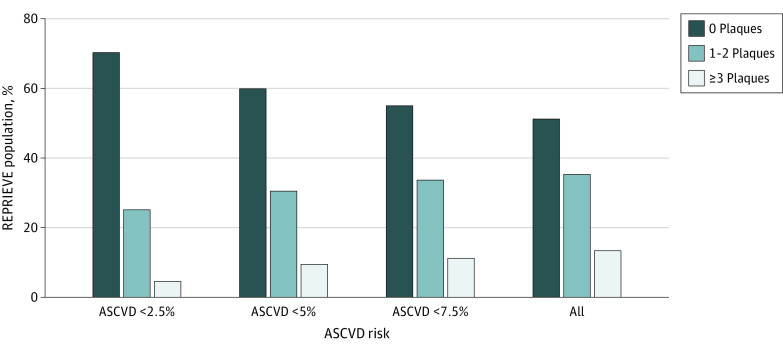
Relative Distribution of Coronary Plaque by Atherosclerotic Cardiovascular Disease (ASCVD) Risk Scores REPRIEVE indicates Randomized Trial to Prevent Vascular Events in HIV.

### Clinical Characteristics of Participants With CAD

Overall, participants with plaque demonstrated higher ASCVD risk than those without plaque, although the absolute difference in median (IQR) PCE risk scores was modest (5.3 [3.3-7.8] vs 3.8 [2.0-5.8], *P* < .001) (Table 3). Participants with plaque were older, more likely to be male and White, to have a family history of premature CVD, to have a history of hypertension, to have increased fasting glucose and LDL-C levels, and tended to smoke. No significant differences were seen in current or nadir CD4 levels or ART duration. No significant differences in class of current ART regimen were seen, although relatively more abacavir and less tenofovir disoproxil fumarate (TDF) use was seen in the entry nucleoside(tide) reverse transcriptase inhibitors (NRTI) regimens of those with plaque and vulnerable plaque. Participants with plaque and vulnerable plaque were also more likely to have been exposed to abacavir. Thymidine exposure was greater among those with plaque, CAC, and high Leaman scores.

**Table 3.** Comparison of Demographic and Clinical Parameters by Presence of Plaque

Parameter	Participants, No./total No. (%)			
	All participants (n = 755)	Coronary plaque		
		None (n = 387)	Present (n = 368)	*P* value
**Demographic characteristics**				
Age, mean (SD), y	50.8 (5.8)	49.5 (5.5)	52.2 (5.8)	<.001
Natal sex				
Women	124 (16.4)	86 (22.2)	38 (10.3)	<.001
Men	631 (83.6)	301 (77.8)	330 (89.7)	
Race				
White	406 (53.8)	185 (47.8)	221 (60.1)	.006
Black or African American	267 (35.4)	158 (40.8)	109 (29.6)	
Asian	10 (1.3)	6 (1.6)	4 (1.1)	
Other^a^	72 (9.5)	38 (9.8)	34 (9.2)	
Ethnicity^b^				
Hispanic or Latinx	182 (24.1)	97 (25.1)	85 (23.1)	.83
Not Hispanic or Latinx	563 (74.6)	285 (73.6)	278 (75.5)	
Unknown	10 (1.3)	5 (1.3)	5 (1.4)	
**Cardiovascular risk factors**				
Smoking status				
Current	181 (24.0)	90 (23.3)	91 (24.8)	.13
Former	235 (31.2)	110 (28.5)	125 (34.1)	
Never	337 (44.8)	186 (48.2)	151 (41.1)	
Substance use				
Current	16 (2.1)	9 (2.3)	7 (1.9)	.05
Former	367 (48.8)	171 (44.4)	196 (53.4)	
Never	369 (49.1)	205 (53.3)	164 (44.7)	
Family history of premature CVD	168 (22.3)	73 (18.9)	95 (26.0)	.02
Hypertension^c^	238 (31.5)	105 (27.1)	133 (36.1)	.01
Diabetes	3 (0.4)	0 (0.0)	3 (0.8)	.12
BMI, mean (SD)	27.3 (4.4)	27.3 (4.6)	27.3 (4.2)	.97
Fasting glucose, mean (SD), mg/dL	93.2 (12.6)	92.2 (11.5)	94.3 (13.7)	.03
eGFR, mean (SD), mL/min/1.73m^2^	88.5 (16.4)	89.4 (16.4)	87.4 (16.4)	.10
Entry fasting lipids, mean (SD)				
LDL-C, mg/dL	107.9 (30.3)	104.0 (29.7)	111.9 (30.4)	<.001
HDL-C, mg/dL	50.5 (18.5)	51.4 (19.1)	49.6 (17.9)	.17
Cardiovascular medications				
Prior statin use	59 (7.8)	20 (5.2)	39 (10.6)	.006
Antihypertensive medication	149 (19.7)	64 (16.5)	85 (23.1)	.03
ASCVD risk score, median (IQR), %	4.5 (2.6-6.8)	3.8 (2.0-5.8)	5.3 (3.3-7.8)	<.001
**HIV parameters**				
Total ART use duration, y				
<5	120 (15.9)	67 (17.3)	53 (14.4)	.19
5-10	199 (26.4)	109 (28.2)	90 (24.5)	
>10	436 (57.8)	211 (54.5)	225 (61.1)	
Entry regimen				
ART regimen by class				
NRTI with INSTI	335 (44.4)	166 (42.9)	169 (45.9)	.77
NRTI with NNRTI	196 (26.0)	107 (27.7)	89 (24.2)	
NRTI with PI	127 (16.8)	67 (17.3)	60 (16.3)	
NRTI sparing	22 (2.9)	10 (2.6)	12 (3.3)	
Other NRTI containing	75 (9.9)	37 (9.6)	38 (10.3)	
Entry NRTI				
Abacavir	128 (17.2)	51 (13.3)	77 (21.3)	.03
TDF	379 (50.9)	209 (54.4)	170 (47.1)	
TAF	211 (28.3)	111 (28.9)	100 (27.7)	
Other	27 (3.6)	13 (3.4)	14 (3.9)	
Protease exposure	464 (61.5)	227 (58.8)	237 (64.4)	.12
TDF exposure	694 (92.0)	360 (93.3)	334 (90.8)	.23
Abacavir exposure	253 (33.6)	109 (28.2)	144 (39.2)	.002
Thymidine exposure	289 (38.4)	126 (32.6)	163 (44.4)	.001
CD4 category, cells/mm^3^				
<350	112 (14.8)	53 (13.7)	59 (16.0)	.27
350-499	148 (19.6)	84 (21.7)	64 (17.4)	
≥500	495 (65.6)	250 (64.6)	245 (66.6)	
Nadir CD4 category, cells/mm^3^				
<50	163 (21.6)	72 (18.6)	91 (24.7)	.28
50-199	218 (28.9)	116 (30.0)	102 (27.7)	
200-349	202 (26.8)	105 (27.1)	97 (26.4)	
≥350	148 (19.6)	79 (20.4)	69 (18.8)	
Unknown	24 (3.2)	15 (3.9)	9 (2.5)	
**Inflammation and immune activation biomarkers, median (IQR)**				
Insulin, μIU/mL	6.7 (4.5-11.7)	6.7 (4.4-11.7)	6.8 (4.7-11.8)	.29
sCD14, ng/mL	1817 (1527-2184)	1838 (1549-2188)	1786 (1468-2176)	.18
sCD163, ng/mL	842 (625-1089)	839 (615-1107)	842 (628-1087)	.67
MCP-1, pg/mL	185 (146-242)	180 (139-229)	194 (155-252)	<.001
IL-6, pg/mL	1.58 (0.99-2.79)	1.45 (0.96-2.60)	1.71 (1.05-3.04)	.008
LpPLA2, ng/mL	130 (92-168)	120 (85-157)	136 (103-177)	<.001
oxLDL, mU/L	53.1 (41.9-69.9)	50.4 (40.4-64.2)	56.6 (45.0-73.3)	<.001
hsCRP, mg/dL	0.18 (0.08-0.36)	0.16 (0.08-0.34)	0.19 (0.08-0.40)	.10
hsCRP categories				
Lower risk, <0.10	219/742 (29.5)	121/380 (31.8)	98/362 (27.1)	.17
Average risk, 0.10-0.30	301/742 (40.6)	155/380 (40.8)	146/362 (40.3)	
Higher risk, 0.31-1.00	161/742 (21.7)	80/380 (21.1)	81/362 (22.4)	
Highest risk, >1.00	61/742 (8.2)	24/380 (6.3)	37/362 (10.2)	

Abbreviations: ART, antiretroviral therapy; ASCVD, atherosclerotic cardiovascular disease; BMI, body mass index (calculated as weight in kilograms divided by height in meters squared); CVD, cardiovascular disease; eGFR, estimated glomerular filtration rate; HDL-C, high density lipoprotein cholesterol; hsCRP, high-sensitivity C-reactive protein; IL-6, interleukin 6; INSTI, integrase strand inhibitor; LDL-C, low-density lipoprotein cholesterol; LpPLA2, lipoprotein-associated phospholipase A2; MCP-1, monocyte chemoattractant protein 1; NNRTI, non–nucleoside reverse transcriptase inhibitor; NRTI, nucleoside reverse transcriptase inhibitor; oxLDL, oxidized LDL; sCD14, soluble CD14; sCD163, soluble CD163; TAF, tenofovir alafenamide; TDF, tenofovir disoproxil fumarate.

SI conversion factors: To convert hsCRP to milligrams per liter, multiply by 10; insulin to picomoles per liter, multiply by 6.945; glucose to millimoles per liter, multiply by 0.0555; and HLD-C and LDL-C to millimoles per liter, multiply by 0.0259.

^a^ Other race includes participants self-identifying as Native or Indigenous to the enrollment region; more than 1 race (with no single race noted as predominant); or of unknown race.

^b^ Ethnicity presented per National Institutes of Health definition.

^c^ Hypertension defined as current diagnosis of hypertension, currently receiving an antihypertensive, or blood pressure greater than 140/90 mm Hg.

### Association of Biomarkers With CAD

Those with coronary plaque had higher levels of IL-6, LpPLA2, oxLDL, and MCP-1 than those without coronary plaque (eg, median [IQR] IL-6 level, 1.71 [1.05-3.04] pg/mL vs 1.45 [0.96-2.60] pg/mL; *P* = .008) (Table 3). Higher levels of IL-6, LpPLA2, oxLDL, and MCP-1 were also seen to varying degrees among those with vulnerable plaque, CAC, and Leaman scores greater than 5 (eTable 4 in Supplement 1). We did not observe a difference in hsCRP concentration categories between the groups with and without plaque, but hsCRP concentrations were higher among those with vulnerable plaque and Leaman scores greater than 5.

### Multivariate Modeling for Coronary Artery Disease Parameters

Among the biomarkers significantly associated with presence of plaque indices in univariate modeling (eTable 5 in Supplement 1), IL-6 and LpPLA2 remained significantly associated in adjusted models, including HIV parameters, the composite ASCVD risk score, and individual ASCVD components (eg, IL-6, adjusted odds ratio, 1.07; 95% CI, 1.02-1.12; *P* = .01) (Table 4). HIV parameters were not significant in the modeling. For other CAD indices, IL-6 was most consistently associated with CAC and vulnerable plaque, whereas hsCRP was consistently associated with Leaman score (eTable 6 in Supplement 1). Similar results were seen in sensitivity analyses, including all biomarkers in each analysis (eTable 7 in Supplement 1). Interaction terms were not significant for biomarkers with age, sex, and race (data not shown). eTable 8 in Supplement 1 includes effects per SD of the log-transformed biomarkers.

**Table 4.** Multivariate Regression Modeling for the Presence of Plaque

Factor	Model 1^a^		Model 2^b^		Model 3^c^	
	aOR (95% CI)	*P* value	aOR (95% CI)	*P* value	aOR (95% CI)	*P* value
**Biomarker**^**d**^						
MCP-1	1.10 (1.00-1.21)	.05	1.06 (0.96-1.17)	.25	1.08 (0.97-1.19)	.15
IL-6	1.06 (1.01-1.11)	.03	1.06 (1.01-1.12)	.01	1.07 (1.02-1.12)	.01
LpPLA2	1.18 (1.09-1.26)	<.001	1.13 (1.04-1.22)	.004	1.11 (1.02-1.20)	.01
oxLDL	1.07 (0.97-1.18)	.18	1.09 (0.98-1.20)	.10	1.01 (0.90-1.15)	.82
**Demographic characteristics and cardiovascular risk**						
ASCVD risk	1.16 (1.10-1.22)	<.001	1.09 (1.01-1.17)	.02	NA	NA
Age	NA	NA	1.07 (1.03-1.11)	<.001	1.10 (1.07-1.14)	<.001
Male	NA	NA	1.70 (1.02-2.83)	.04	2.37 (1.49-3.78)	<.001
Race						
White	NA	NA	1 [Reference]	NA	1 [Reference]	NA
Black	NA	NA	0.63 (0.42-0.96)	.03	0.64 (0.43-0.96)	.03
Asian	NA	NA	1.39 (0.35-5.51)	.64	1.63 (0.40-6.65)	.50
Other	NA	NA	0.98 (0.57-1.70)	.95	1.04 (0.59-1.82)	.89
LDL-C	NA	NA	NA	NA	1.01 (1.00-1.02)	.02
Hypertension	NA	NA	NA	NA	1.54 (1.08-2.18)	.02
Current smoking	NA	NA	NA	NA	1.70 (1.13-2.54)	.01
**HIV parameters**						
Total ART use duration, y						
<5	1 [Reference]	NA	1 [Reference]	NA	1 [Reference]	NA
5-10	0.84 (0.51-1.37)	.48	0.88 (0.53-1.46)	.61	0.78 (0.47-1.31)	.36
>10	1.03 (0.65-1.62)	.90	0.98 (0.62-1.55)	.93	0.92 (0.58-1.47)	.74
CD4 category, cells/mm^3^						
<350	1 [Reference]	NA	1 [Reference]	NA	1 [Reference]	NA
350-499	0.83 (0.49-1.42)	.50	0.83 (0.48-1.44)	.51	0.82 (0.47-1.43)	.48
≥500	0.97 (0.60-1.57	.91	0.99 (0.60-1.61)	.96	0.97 (0.59-1.59)	.89
Nadir CD4 category, cells/mm^3^						
<50	1 [Reference]	NA	1 [Reference]	NA	1 [Reference]	NA
50-199	0.62 (0.40-0.96)	.03	0.57 (0.36-0.89)	.01	0.54 (0.34-0.85)	.008
200-349	0.72 (0.45-1.14)	.16	0.63 (0.39-1.01)	.06	0.66 (0.40-1.07)	.09
≥350	0.69 (0.40-1.16)	.16	0.66 (0.38-1.12)	.13	0.66 (0.38-1.15)	.14
Unknown	0.54 (0.21-1.40	.20	0.45 (0.17-1.20)	.11	0.42 (0.15-1.13)	.09

Abbreviations: ART, antiretroviral therapy; ASCVD, atherosclerotic cardiovascular disease; IL-6, interleukin 6; LDL-C, low density lipoprotein cholesterol; LpPLA2, lipoprotein-associated phospholipase A2; MCP-1, monocyte chemoattractant protein 1; NA, not applicable; oxLDL, oxidized LDL.

^a^ All biomarkers that were significant in univariate analysis, ASCVD risk, HIV Parameters (ART duration, CD4, nadir CD4).

^b^ Same variables as model 1 plus age, sex, and race.

^c^ Same variables as model 2 except ASCVD risk but including LDL-C level, hypertension, and current smoking.

^d^ All biomarkers log transformed using log2 divided by 0.32192809, to give effects per 25% increase of biomarker value.

## Discussion

CVD is a major source of morbidity and mortality among PWH receiving ART, but little is known regarding the extent of CAD and key associated factors in those with low to moderate traditional cardiovascular risk. This study, performed in a large primary prevention cohort of relatively young patients, expands our understanding of CAD in HIV, demonstrating a substantial prevalence of coronary atherosclerosis, including vulnerable plaque. Markers of innate immune activation and arterial inflammation are associated with CAD in this group with well-controlled HIV disease.

Studies to date have shown excess CAD occurring at a younger age among PWH.^25^ Two key studies using CTA suggested an increased prevalence of plaque in this population. However, such studies have often been limited to men^13^ and/or have been relatively small.^11,14^ To our knowledge, prior studies have not assessed plaque using CTA in a prospectively recruited asymptomatic primary prevention cohort with low to moderate ASCVD risk, assessed in the current era of modern ART, using the gold standard ACC/AHA pooled cohort equation (PCE) for risk calibration. This question is of critical importance to the large group of relatively young PWH at risk for but without known CVD. In this regard, these baseline data from the mechanistic substudy of REPRIEVE provide useful information on the degree and type of CAD among this primary prevention group. Plaque was seen in nearly 50% and CAC in 35% of our population, despite a mean age of 51 years and a median ASCVD risk of 4.5%. Plaque characteristics in this group suggest a low prevalence of significant stenosis, but vulnerable plaque characteristics were seen in nearly one-quarter. It will be critical in future studies to determine how this unique plaque phenotype relates to major adverse cardiovascular events over time.

Comparator data from other primary prevention populations with low to moderate risk are available for CAC score, but very limited data are available for more detailed plaque characteristics. Data from the Framingham Heart Study and Cardia Study Cohorts showed a prevalence of CAC scores greater than 0 of 30% and 28%, respectively, in patients aged 50 years, either free of cardiac disease or with a low Framingham Risk score.^26,27^ However, the CAC score is only a single measure of coronary atherosclerosis, and our study also assessed noncalcified plaque and vulnerable plaque. In this regard, our study showed vulnerable plaque in 23% of participants compared with 15% in the much older PROMISE population of symptomatic patients with higher ASCVD risk, assessed in an identical fashion with contrast-enhanced CT by the same imaging core.^19^ In contrast, a CAC score greater than 0 was observed in 65% of the PROMISE population but 35% of our study population, consistent with more advanced traditional risk patterns of the PROMISE population.^28^

In assessing the association of plaque with ASCVD risk categories, we identified significant trends of increased plaque across increasing risk categories. Participants with plaque were older, more likely to be male, less often Black or African American individuals, and more often White individuals. Participants with plaque had a higher prevalence of hypertension and a family history of premature CAD. LDL-C and glucose levels were higher among those with plaque, as was overall ASCVD risk, although risk scores in those with plaque were still relatively low. Overall, diabetes rates were low, based on the primary prevention focus of the cohort, and not different between groups. Thus, CAD indices do increase with increasing traditional risk factors, even among PWH with low to moderate traditional risk scores, indicating the potential importance of risk modification in this group, not often targeted for prevention strategies.

Most HIV parameters did not differ in terms of CAD indices. Abacavir was administered more often and TDF less often in the entry NRTI regimen among those with plaque vs without. In addition, a greater percentage of those with plaque vs without plaque had prior exposure to abacavir. Abacavir use has been associated with increased myocardial infarction in some studies^29^ and shown to stimulate endothelial cell activation^30^ and platelet activation and/or reactivity.^31^ The Swiss HIV cohort demonstrated an association of mixed plaque with abacavir exposure,^32^ but this association was not observed in the Multicenter AIDS Cohort study.^33^ REPRIEVE is not a randomized trial of ART, limiting causal inference. Further studies are needed to assess the association of specific NRTI therapies with plaque progression.

In this study, we assessed specific biomarkers hypothesized to play a role in premature CAD among PWH. In adjusted analyses, controlling for ASCVD risk, the inflammatory and immune markers LpPLA2 and IL-6 were associated with plaque presence independent of traditional risk factors. IL-6 was also associated with CAC and vulnerable plaque. In contrast, hsCRP, a marker of general inflammation, was significant in the adjusted model for Leaman score, an index of plaque composition and extent. Prior studies have shown persistent immune activation even among PWH with good virologic control.^4^ IL-6 is an important inflammatory cytokine involved in innate immune function, induced by toll-like receptors on neutrophils and monocytes, associated with the incident cardiovascular events in PWH receiving suppressive ART.^34^ We also saw a strong signal for LpPLA2, a marker of arterial inflammation involved in the hydrolysis of oxLDL and the production of proinflammatory mediators of plaque formation. These data build on prior studies demonstrating increased LpPLA2^35,36^ and aortic inflammation in PWH. Data from REPRIEVE connect inflammation and immune activation to CAD in a large study of PWH, under good virologic control, with low traditional risk.

### Limitations

This study has strengths but also some limitations. These data are from a large, primary prevention cohort of PWH at low to moderate traditional CVD risk, prospectively recruited across multiple sites in the US, with a high percentage of relatively young participants, participants from diverse racial and ethnic groups, and women. CAD phenotypes may differ in other regions. The purpose of this baseline analysis of the REPRIEVE mechanistic study was to define prevalence and extent of CAD across traditional risk strata and assess associations with critical immune and inflammatory biomarkers, rather than comparing them with a control population. The cross-sectional nature of these baseline data limits conclusions on causality with respect to specific inflammatory pathways and plaque.

## Conclusions

This study found a substantial prevalence of CAD even in young PWH with low traditional ASCVD risk. Key markers of innate immune activation and arterial inflammation were associated with CAD in this group with well-controlled HIV disease, independent of traditional risk factors. Further study of this cohort will help to determine the effects of statin therapy to modulate these pathways and reduce plaque in this population.

## Notes

Supplement 1. **eFigure 1. **Flowchart **eFigure 2. **Plaque by CVD Risk Category **eMethods.** Supplemental Methods **eTable 1. ** REPRIEVE (A5332) Enrollment Criteria **eTable 2. **A5333s Clinical Research Site Listing **eTable 3. **Assay Characteristics of the Biomarkers **eTable 4. **Comparison of Demographic and Clinical Parameters by Coronary Artery Disease Status **eTable 5. **Full Univariate Regression Modeling for Coronary Artery Disease Parameters **eTable 6.** Multivariate Regression Modeling for Coronary Artery Disease Parameters **eTable 7.** Multivariate Regression Modeling for Coronary Artery Disease Parameters Including All Biomarkers and Covariates **eTable 8. **Multivariate Regression Modeling for Coronary Artery Disease Parameters per SD of Log Transformed Values

Supplement 2. Nonauthor Collaborators
