# Supplementary material for: Assessment of Coronary Artery Disease With Computed Tomography Angiography and Inflammatory and Immune Activation Biomarkers Among Adults With HIV Eligible for Primary Cardiovascular Prevention
Source: JAMA Netw Open. 2021 Jun 29;4(6):e2114923. doi: 10.1001/jamanetworkopen.2021.14923 (PMC8243232; doi:10.1001/jamanetworkopen.2021.14923)
Supplement: Supplement 1. — eFigure 1. Flowchart eFigure 2. Plaque by CVD Risk Category eMethods. Supplemental Methods eTable 1. REPRIEVE (A5332) Enrollment Criteria eTable 2. A5333s Clinical Research Site Listing eTable 3. Assay Characteristics of the Biomarkers eTable 4. Comparison of Demographic and Clinical Parameters by Coronary Artery Disease Status eTable 5. Full Univariate Regression Modeling for Coronary Artery Disease Parameters eTable 6. Multivariate Regression Modeling for Coronary Artery Disease Parameters eTable 7. Multivariate Regression Modeling for Coronary Artery Disease Parameters Including All Biomarkers and Covariates eTable 8. Multivariate Regression Modeling for Coronary Artery Disease Parameters per SD of Log Transformed Values [file jamanetwopen-e2114923-s001.pdf]

Supplemental Online Content

Hoffmann U, Lu MT, Foldyna B, et al; REPRIEVE trial. Assessment of coronary artery disease with computed tomography angiography and inflammatory and immune activation biomarkers among adults with HIV eligible for primary cardiovascular prevention. *JAMA Netw Open.* 2021;4(6):e2114923. doi:10.1001/jamanetworkopen.2021.14923

eFigure 1. Flowchart

eFigure 2. Plaque by CVD Risk Category

eMethods. Supplemental Methods

eTable 1. REPRIEVE (A5332) Enrollment Criteria

eTable 2. A5333s Clinical Research Site Listing

eTable 3. Assay Characteristics of the Biomarkers

eTable 4. Comparison of Demographic and Clinical Parameters by Coronary Artery Disease Status

eTable 5. Full Univariate Regression Modeling for Coronary Artery Disease Parameters

eTable 6. Multivariate Regression Modeling for Coronary Artery Disease Parameters

eTable 7. Multivariate Regression Modeling for Coronary Artery Disease Parameters Including All Biomarkers and Covariates

eTable 8. Multivariate Regression Modeling for Coronary Artery Disease Parameters per SD of Log Transformed Values

This supplemental material has been provided by the authors to give readers additional information about their work.

© 2021 Hoffmann U et al. *JAMA Network Open.*

eFigure 1. Flowchart

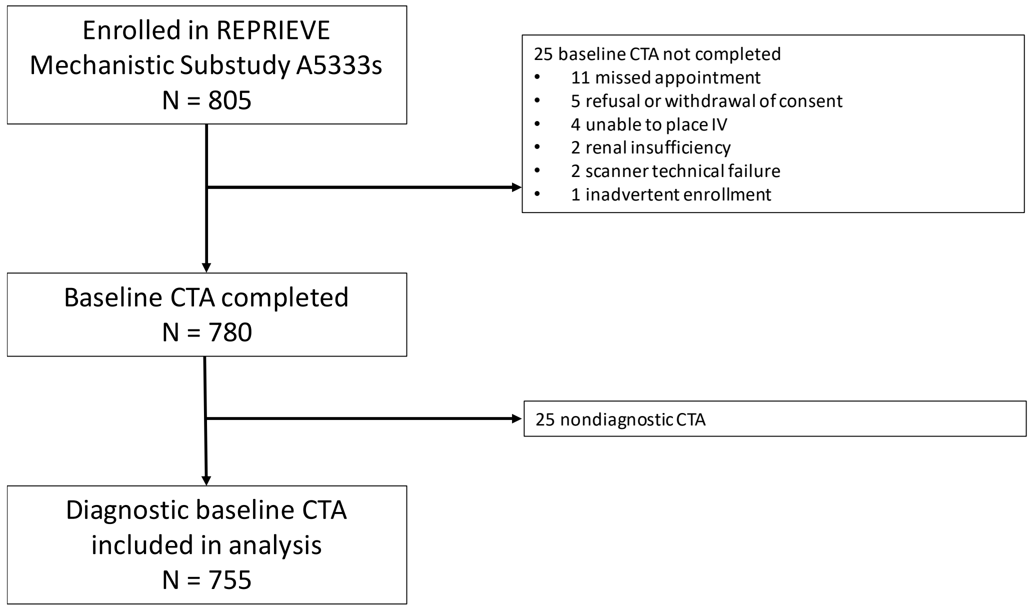

Abbreviations: CTA, computed tomography angiography.

© 2021 Hoffmann U et al. *JAMA Network Open.*

eFigure 2. Plaque by CVD Risk Category

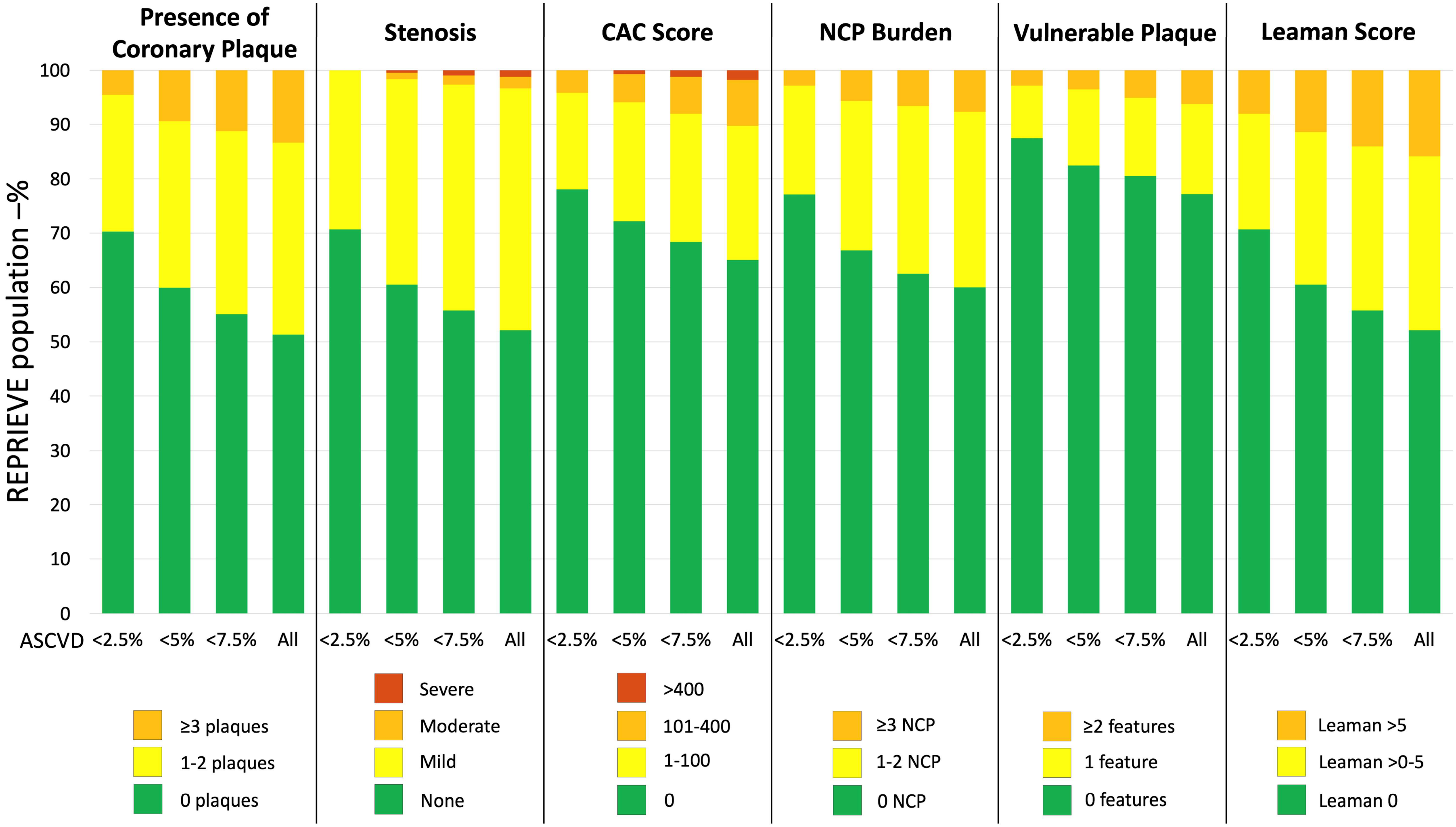

Bar charts demonstrating relative distribution of CAD indices by ASCVD risk scores. Abbreviations: ASCVD, atherosclerotic cardiovascular disease; CAC, coronary artery calcium; NCP, noncalcified plaque.

© 2021 Hoffmann U et al. *JAMA Network Open.*

eMethods. Supplemental Methods

CT Acquisition

Sublingual nitroglycerin and oral and/or intravenous beta-blocker were administered for vasodilation and heart rate control, respectively. Non-contrast prospectively ECG-triggered cardiac CT for evaluation of the coronary artery calcium score, followed by an intravenous contrast-enhanced prospectively ECG-triggered or retrospectively ECG-gated coronary CT angiogram. Coronary CTA images were reconstructed at a slice thickness <1 mm with a field of view <250 mm to optimize voxel resolution.

Imputation of CRP

As per Quest, the following categories were used to stratify hsCRP, <1 mg/L (lower relative cardiovascular risk, 1.0-3.0 mg/L, average relative cardiovascular risk, and 3.1-10 mg/L, higher relative cardiovascular risk. To utilize CRP in continuous modeling, values censored <0.3mg/L (n=25) and > 10 mg/L (n=61) were randomly imputed using a log10 uniform distribution.

© 2021 Hoffmann U et al. *JAMA Network Open.*

eTable 1. REPRIEVE (A5332) Enrollment Criteria

INCLUSION CRITERIA	EXCLUSION CRITERIA
**Men and women age ≥40 and ≤75 years of age**<br>**Documentation of HIV-1 infection**<br>**Combination ART for at least 180 d prior to study entry**<br>**CD4+ cell count N100 cells/mm3**<br>**Fasting LDL-C as follows:**<br>**If ASCVD risk score* <7.5%, LDL-C must be <190 mg/dL**<br>**If ASCVD risk score ≥7.5% and ≤10%, LDL-C must be <160 mg/dL**<br>**If ASCVD risk score >10% and ≤15%, LDL-C must be <130 mg/dL**<br>***ASCVD risk score calculated using the 2013 ACC/AHA Pooled Cohort Equation, including variables for age, sex, race, blood pressure, diabetes, smoking, cholesterol, LDL-C, HDL-C, hypertension therapy, statin use and aspirin use.**<br>**Fasting triglycerides <500 mg/dL**<br>**Hemoglobin ≥8 g/dL for female participants and ≥9 g/dL for male participants**<br>**eGFR ≥60 mL/min/1.73 m^2 or CrCl ≥60 mL/min**<br>**ALT ≤2.5 × ULN**<br>**For persons with known chronic active HBV or HCV, calculated Fibrosis-4 score must be ≤3.25**<br>**Female participants of reproductive potential must have a negative serum or urine pregnancy test result**<br>**For women of reproductive potential, willingness to use contraceptives as described in the product information for pitavastatin**<br>**Ability and willingness of participant or legal representative to provide written informed consent**	Clinical ASCVD, as defined by 2013 ACC/AHA guidelines<br>Current diabetes mellitus if LDL-C ≥70 mg/dL<br>10-y ASCVD risk score estimated by Pooled Cohort Equation >15%<br>Active cancer within 12 m prior to study entry<br>Known decompensated cirrhosis<br>History of myositis or myopathy with active disease in the 180 d prior to study entry<br>Known untreated symptomatic thyroid disease<br>History of allergy or severe adverse reaction to statins<br>Use of specific immunosuppressants or immunomodulatory agents in the 30 d prior to study entry<br>Current use of erythromycin, colchicine, or rifampin<br>Use of any statin drugs, gemfibrozil, or PCSK9 inhibitors in the 90d prior to study entry<br>Current use of an investigational new drug that would be contraindicated<br>Serious illness or trauma requiring systemic treatment or hospitalization in the 30 d prior to study entry<br>Known active or recent (not fully resolved within 30 d prior to study entry) systemic bacterial, fungal, parasitic, or viral infections (except HIV, HBV, HPV, or HCV)<br>Current breastfeeding<br>Alcohol or drug use that, in the opinion of the site investigator, would interfere with completion of study procedures<br>Other medical, psychiatric, or psychological condition that, in the opinion of the site investigator, would interfere with completion of study procedures and or adherence to study drug

Abbreviations: ACC/AHA, American College of Cardiology/American Heart Association; ALT, alanine transaminase; ART, antiretroviral therapy; ASCVD, atherosclerotic cardiovascular disease; CAC, coronary artery calcium; CrCl, creatinine clearance; eGFR, estimated glomerular filtration rate; HBV, hepatitis B virus; HBC, hepatitis C virus; HDL-C, high density lipoprotein cholesterol; HIV, Human immunodeficiency virus; HPV, human papilloma virus; LDL-C, low density lipoprotein cholesterol; NCP, noncalcified plaque; PCSK-9, proprotein convertase subtilisin/kexin type 9; ULN, upper liit of normal.

© 2021 Hoffmann U et al. *JAMA Network Open.*

eTable 2. A5333s Clinical Research Site Listing

**Clinical research sites**
**Massachusetts General Hospital (MGH) CRS 101**
**Brigham and Women's Hospital Therapeutics CRS 107**
**Johns Hopkins University CRS 201**
**UCLA CARE Center CRS 601**
**Harbor UCLA CRS 603**
**UCSD Antiviral Research Center CRS 701**
**UCSF HIV/AIDS CRS 801**
**University of Pittsburgh CRS 1001**
**University of Southern California CRS 1201**
**University of Washington AIDS CRS 1401**
**Washington University Therapeutics (WT) CRS 2101**
**Ohio State University CRS 2301**
**Cincinnati CRS 2401**
**Case CRS 2501**
**Northwestern University CRS 2701**
**Rush University CRS 2702**
**The Miriam Hosp (TMH) CRS 2951**
**Chapel Hill CRS 3201**
**Greensboro CRS 3203**
**Vanderbilt Therapeutics (VT) CRS 3652**
**Puerto Rico AIDS Clinical Trials Unit CRS 5401**
**University of Colorado Hospital CRS 6101**
**Penn Therapeutics CRS 6201**
**Weill Cornell Uptown CRS 7803**
**Weill Cornell Chelsea CRS 7804**
**Columbia Physicians and Surgeons CRS 30329**
**Houston AIDS Research Team CRS 31473**
**New Jersey Medical School-Clinical Research Ctr. CRS 31786**
**University of Rochester Adult HIV Therapeutic Network CRS 31787**
**Alabama CRS 31788**
**Mount Sinai Clinical and Translational Research Center CRS 31883**

© 2021 Hoffmann U et al. *JAMA Network Open.*

eTable 3. Assay Characteristics of the Biomarkers

Marker	Company	Catalog#	Minimal Detectable Dose (MDD) and Range	Conc.	Intra-Assay Variability	Inter-Assay Variability
HSIL-6	R&D	HS600C	MDD ranged from 0.007–0.090 pg/mL	pg/mL	3.6–4.7%	3.9–10.8%
sCD14	R&D	DC140	MDD is typically less than 125 pg/mL	ng/mL	4.8–6.4%	4.8–7.4%
MCP-1	R&D	DCP00	MDD ranged from 0.57–10.0 pg/mL	pg/mL	4.2–5.9%	4.5–5.9%
LpPLA2	R&D	DPLG70	MDD ranged from 0.025–0.284 ng/mL	ng/mL	2.3–6.8%	5.2–9.6%
sCD163	IQ Products	IQP-383	Detection level of the assay is 0.23 ng/mL	ng/mL	3–6%	5–8%
oxLDL	Mercodia	10-1143-01	Detection limit is 0.6 mU/L	mU/L	5.5–7.3%	7.4–8.3%
hsCRP	Quest	10124	Reportable range 0.3–10.0 mg/L	mg/L	0.71%	1.01%
Insulin	Quest	561	Reportable range 1–3,000 uIU/mL	uIU/mL	2.80%	2.86%

Abbreviations: hsCRP, high sensitivity C-reactive protein; IL-6, interleukin-6; LpPLA2, lipoprotein-associated phospholipase A2; MCP-1, monocyte chemoattractant protein-1; oxLDL, oxidized LDL; sCD14, soluble CD14; sCD163, soluble CD163.

© 2021 Hoffmann U et al. *JAMA Network Open.*

eTable 4. Comparison of Demographic and Clinical Parameters by Coronary Artery Disease Status

–n/N (%), mean±SD, or median [25%–75%]		Calcium Score (CAC)			Vulnerable Plaque (VP)			Leaman Score (LS)		
	**All participants** N=755 (100%)	**CAC=0** N=467 (65.0%)	**CAC>0** N=251 (35.0%)	**p-value**	**No VP** N=583 (77.2%)	**Any VP** N=172 (22.8%)	**p-value**	**LS ⩽5** N=625 (84.1%)	**LS >5** N=118 (15.9%)	**p-value**
**Demographics**										
Age –years	50.8±5.8	49.9±5.6	52.6±5.9	<0.01	50.4±5.7	52.4±5.9	<0.01	50.4±5.8	53.0±5.5	<0.01
Natal sex				<0.01			<0.01			0.02
Women	124 (16.4)	93 (19.9)	28 (11.2)		107 (18.4)	17 (9.9)		111 (17.8)	11 (9.3)	
Men	631 (83.6)	374 (80.1)	223 (88.8)		476 (81.7)	155 (90.1)		514 (82.2)	107 (90.7)	
Race				0.06			0.24			0.22
White	406 (53.8)	237 (50.8)	152 (60.6)		306 (52.5)	100 (58.1)		327 (52.3)	70 (59.3)	
Black or African American	267 (35.4)	177 (37.9)	72 (28.7)		212 (36.4)	55 (32.0)		230 (36.8)	35 (29.7)	
Asian	10 (1.3)	6 (1.3)	4 (1.6)		6 (1.0)	4 (2.3)		7 (1.1)	3 (2.5)	
Other	72 (9.5)	47 (10.1)	23 (9.2)		59 (10.1)	13 (7.6)		61 (9.8)	10 (8.5)	
Ethnicity				0.05			0.74			0.06
Hispanic or Latinx	182 (24.1)	126 (27.0)	49 (19.5)		139 (23.8)	43 (25.0)		157 (25.1)	20 (17.0)	
Not Hispanic or Latinx	563 (74.6)	334 (71.5)	200 (79.7)		437 (75.0)	126 (73.3)		461 (73.8)	95 (80.5)	
Unknown	10 (1.3)	7 (1.5)	2 (0.8)		7 (1.2)	3 (1.7)		7 (1.1)	3 (2.5)	
**Cardiovascular risk factors**										
Smoking Status				0.13			0.01			0.01
Current	181 (24.0)	106 (22.7)	63 (25.2)		131 (22.5)	50 (29.2)		142 (22.8)	36 (30.5)	
Former	235 (31.2)	138 (29.6)	87 (34.8)		174 (29.9)	61 (35.7)		189 (30.3)	44 (37.3)	
Never	337 (44.8)	223 (47.8)	100 (40.0)		277 (47.6)	60 (35.1)		292 (46.9)	38 (32.2)	
Substance use				<0.01			0.03			0.21
Current	16 (2.1)	11 (2.4)	5 (2.0)		9 (1.6)	7 (4.1)		15 (2.4)	1 (0.9)	
Former	367 (48.8)	209 (44.9)	142 (56.8)		276 (47.5)	91 (53.2)		295 (47.4)	66 (55.9)	
Never	369 (49.1)	246 (52.8)	103 (41.2)		296 (51.0)	73 (42.7)		312 (50.2)	51 (43.2)	
Family history of premature CVD	168 (22.3)	97 (20.8)	62 (24.9)	0.03	128 (22.0)	40 (23.5)	0.41	136 (21.8)	29 (24.8)	0.02
Hypertension	238 (31.5)	132 (28.3)	95 (37.9)	<0.01	172 (29.5)	66 (38.4)	0.03	186 (29.8)	45 (38.1)	0.08
Diabetes	3 (0.4)	1 (0.2)	2 (0.8)	0.28	2 (0.3)	1 (0.6)	0.54	1 (0.2)	2 (1.7)	0.07
BMI –kg/m^2	27.3±4.4	27.3±4.5	27.3±4.0	0.97	27.4±4.4	27.1±4.2	0.58	27.3±4.4	27.2±4.2	0.77
Fasting glucose –mg/dL	93.2±12.6	92.5±12.2	94.0±12.9	0.14	92.6±12.2	95.3±13.8	0.02	93.2±12.8	93.6±12.4	0.72
eGFR –mL/min/1.73m^2	88.5±16.4	88.5±16.4	87.4±16.2	0.38	88.7±16.7	87.5±15.4	0.38	88.3±16.4	89.4±16.2	0.47
**Entry fasting lipids**										
LDL-C –mg/dL	107.9±30.3	107.0±31.0	110.4±30.1	0.16	106.5±30.1	112.5±30.3	0.02	106.8±30.2	110.7±29.0	0.18
HDL-C –mg/dL	50.5±18.5	50.6±18.5	50.7±18.8	0.98	50.3±18.8	51.4±17.6	0.48	51.0±19.1	48.3±15.2	0.10
**Cardiovascular medications**										
Prior statin use	59 (7.8)	31 (6.6)	26 (10.4)	0.08	41 (7.0)	18 (10.5)	0.15	46 (7.4)	11 (9.3)	0.45
Antihypertensive medication	149 (19.7)	79 (16.9)	63 (25.1)	0.01	105 (18.0)	44 (25.6)	0.04	114 (18.2)	30 (25.4)	0.08
**Median ASCVD risk score**	4.5 [2.6–6.8]	3.9 [2.2-6.1]	5.4 [3.3–7.9]	<0.01	4.2 [2.4–6.3]	5.6 [3.9-8.0]	<0.01	4.2 [2.4–6.4]	5.6 [4.0–8.0]	<0.01
**HIV parameters**										
Total ART use duration –years				0.25			0.81			0.06
<5	120 (15.9)	80 (17.1)	36 (14.3)		95 (16.3)	25 (14.5)		100 (16.0)	20 (17.0)	
5–10	199 (26.4)	127 (27.2)	59 (23.5)		155 (26.6)	44 (25.6)		175 (28.0)	21 (17.8)	
>10	436 (57.8)	260 (55.7)	156 (62.2)		333 (57.1)	103 (59.9)		350 (56.0)	77 (65.3)	
Entry regimen										

© 2021 Hoffmann U et al. *JAMA Network Open.*

ART Regimen by Class				0.80			0.36			0.39
NRTI + INSTI	335 (44.4)	206 (44.1)	117 (46.6)		251 (43.1)	84 (48.8)		277 (44.3)	53 (44.9)	
NRTI + NNRTI	196 (26.0)	123 (26.3)	60 (23.9)		152 (26.1)	44 (25.6)		158 (25.3)	37 (31.4)	
NRTI + PI	127 (16.8)	81 (17.3)	41 (16.3)		104 (17.8)	23 (13.4)		110 (17.6)	13 (11.0)	
NRTI-sparing	22 (2.9)	11 (2.4)	9 (3.6)		15 (2.6)	7 (4.1)		19 (3.0)	3 (2.5)	
Other NRTI-containing	75 (9.9)	46 (9.9)	24 (9.6)		61 (10.5)	14 (8.1)		61 (9.8)	12 (10.2)	
Entry NRTI				0.30			<0.01			0.09
ABC	128 (17.2)	73 (15.8)	50 (20.3)		85 (14.7)	43 (25.8)		96 (15.6)	29 (25.0)	
TDF	379 (50.9)	239 (51.7)	120 (48.8)		309 (53.5)	70 (41.9)		318 (51.5)	57 (49.1)	
TAF	211 (28.3)	136 (29.4)	65 (26.4)		165 (28.6)	46 (27.5)		180 (29.2)	26 (22.4)	
Other	27 (3.6)	14 (3.0)	11 (4.5)		19 (3.3)	8 (4.8)		23 (3.7)	4 (3.5)	
Protease exposure	464 (61.5)	273 (58.6)	167 (66.5)	0.04	358 (61.5)	106 (61.6)	1.00	388 (62.2)	69 (58.5)	0.47
TDF exposure	694 (92.0)	432 (92.7)	228 (90.8)	0.39	542 (93.1)	152 (88.4)	0.05	578 (92.6)	107 (90.7)	0.45
Abacavir exposure	253 (33.6)	148 (31.8)	97 (38.8)	0.07	181 (31.1)	72 (42.1)	0.01	201 (32.3)	46 (39.0)	0.17
Thymidine exposure	289 (38.4)	159 (34.1)	111 (44.4)	<0.01	219 (37.6)	70 (40.9)	0.47	225 (36.1)	58 (49.2)	0.01
CD4 category				0.47			0.01			0.08
CD4 <350	112 (14.8)	67 (14.4)	43 (17.1)		88 (15.1)	24 (14.0)		88 (14.1)	22 (18.6)	
CD4 350–499	148 (19.6)	88 (18.8)	51 (20.3)		127 (21.8)	21 (12.2)		130 (20.8)	15 (12.7)	
CD4 ⩾500	495 (65.6)	312 (66.8)	157 (62.6)		368 (63.1)	127 (73.8)		407 (65.1)	81 (68.6)	
Nadir CD4 category				0.11			0.75			0.32
Nadir CD4 <50	163 (21.6)	89 (19.1)	66 (26.3)		123 (21.1)	40 (23.3)		127 (20.3)	32 (27.1)	
Nadir CD4 50–199	218 (28.9)	136 (29.1)	70 (27.9)		170 (29.2)	48 (27.9)		185 (29.6)	29 (24.6)	
Nadir CD4 200–349	202 (26.8)	124 (26.6)	69 (27.5)		160 (27.4)	42 (24.4)		173 (27.7)	27 (22.9)	
Nadir CD4 ⩾350	148 (19.6)	102 (21.8)	39 (15.5)		110 (18.9)	38 (22.1)		119 (19.0)	27 (22.9)	
Unknown	24 (3.2)	16 (3.4)	7 (2.8)		20 (3.4)	4 (2.3)		21 (3.4)	3 (2.5)	
**Inflammation & Immune Activation Biomarkers**										
Insulin –uIU/mL	6.7 [4.5–11.7]	6.8 [4.4–11.8]	6.7 [4.5–10.9]	0.88	6.8 [4.5–11.8]	6.7 [4.5–11.3]	0.96	6.8 [4.4–11.5]	6.7 [4.7–12.0]	0.45
sCD14 –ng/mL	1817 [1527–2184]	1827 [1537–2166]	1806 [1456–2198]	0.94	1824 [1529–2173]	1806 [1475–2248]	0.56	1813 [1526–2169]	1822 [1542–2310]	0.56
sCD163 –ng/mL	842 [625–1089]	834 [625–1075]	846 [628–1088]	0.58	845 [630–1108]	833 [614–1076]	0.51	832 [615–1082]	861 [656–1097]	0.26
MCP-1 –pg/mL	185 [146–242]	183 [142–230]	194 [149–253]	0.02	183 [142–239]	195 [156–247]	0.02	183 [145–234]	194 [150–257]	0.14
IL-6 –pg/mL	1.58 [0.99–2.79]	1.50 [0.96–2.59]	1.79 [1.05–3.17]	0.01	1.52 [0.96–2.72]	1.90 [1.13–3.17]	<0.01	1.52 [0.99–2.64]	1.91 [1.02–3.33]	0.03
LpPLA2 –ng/mL	130 [92–168]	125 [90–163]	135 [102–176]	0.02	131 [92–167]	129 [91–170]	0.81	126 [90–164]	133 [105–178]	0.04
oxLDL –mU/L	53.1 [41.9–69.9]	52.3 [41.4–68.1]	55.3 [43.7–72.4]	0.07	51.9 [41.3–68.1]	57.6 [45.6–73.2]	<0.01	52.5 [41.5–69.4]	53.5 [44.2–69.1]	0.51
hsCRP –mg/L	1.75 [0.80–3.60]	1.70 [0.80–3.50]	1.80 [0.80–4.00]	0.57	1.70 [0.80–3.50]	2.20 [1.10–4.20]	0.02	1.70 [0.80–3.30]	2.35 [0.95–5.10]	0.02
hsCRP categories				0.24			0.09			0.01
Lower risk <1.0	219/742 (29.5)	136/458 (29.7)	73/247 (29.6)		182/575 (31.7)	37/167 (22.2)		190/614 (30.9)	29/116 (25.0)	
Average risk 1.0–3.0	301/742 (40.6)	190/458 (41.5)	96/247 (38.9)		230/575 (40.0)	71/167 (42.5)		253/614 (41.2)	42/116 (36.2)	

© 2021 Hoffmann U et al. *JAMA Network Open.*

Higher risk 3.1–10.0	161/742 (21.7)	102/458 (22.3)	51/247 (20.7)		119/575 (20.7)	42/167 (25.2)		132/614 (21.5)	27/116 (23.3)	
Highest risk >10.0	61/742 (8.2)	30/458 (6.6)	27/247 (10.9)		44/575 (7.7)	17/167 (10.2)		39/614 (6.4)	18/116 (15.5)	

Other' race includes participants self-identifying as: native or indigenous to the enrollment region; more than one race (with no single race noted as predominant); or of unknown race. Ethnicity presented per NIH definition. Hypertension defined as current diagnosis of hypertension, or currently on an antihypertensive, or blood pressure > 140/90 mm Hg.

Abbreviations: ABC, abacavir; ASCVD, atherosclerotic cardiovascular disease; ART, antiretroviral therapy; BMI, body-mass-index; HDL-C, high density lipoprotein cholesterol; HIV, Human immunodeficiency virus; hsCRP, high sensitivity C-reactive protein; INSTI, integrase strand inhibitor; IL-6, interleukin-6; LDL-C, low density lipoprotein cholesterol; LLQ, lower limit of quantification; LpPLA2, lipoprotein-associated phospholipase A2; MCP-1, monocyte chemoattractant protein-1; NNRTI, non-nucleoside reverse transcriptase inhibitor; NRT, nucleoside reverse transcriptase inhibitor; oxLDL, oxidized LDL; sCD14, soluble CD14; sCD163, soluble CD163; TAF, tenofovir alafenamide; TDF, tenofovir disoproxil fumarate.

All statistics are calculated out of participants with data collected. Missing data: Smoking status (n=2); Substance use (n=3); Family history of premature CVD (n=24); Fasting glucose (n=10); LDL-C (n=13); HDL-C (n=7); HIV-1 RNA (n=10); Protease exposure.(n=1); TDF exposure (n=1); Abacavir exposure (n=2); Thymidine exposure (n=2); Insulin (n=13; sCD14 (n=12); sCD163 (n=12); MCP-1 (n=13); IL-6 (n=12); LpPLA2 (n=12); oxLDL (n=12); hsCRP (n=13).

For glucose, conversion from mg/dL to mmol/L is 0.0555; for LDL-C, conversion from mg/dL to mmol/L is 0.0259; for HDL-C, conversion from mg/dL to mmol/L is 0.0259.

© 2021 Hoffmann U et al. *JAMA Network Open.*

eTable 5. Full Univariate Regression Modeling for Coronary Artery Disease Parameters

a) Presence of Plaque

	Univariate Models*		
**Biomarker#**	**OR**	**95%CI**	**p-value**
Insulin	1.04	0.99 - 1.08	0.12
sCD14	0.91	0.81 - 1.02	0.12
sCD163	1.03	0.95 - 1.11	0.48
MCP-1	1.17	1.07 - 1.27	<0.01
IL-6	1.06	1.02 - 1.11	<0.01
LpPLA2	1.17	1.10 - 1.26	<0.01
OxLDL	1.17	1.07 - 1.27	<0.01
hsCRP	1.03	1.00 - 1.07	0.08

b) Calcium Score >0

	Univariate Models*		
**Biomarker#**	**OR**	**95%CI**	**p-value**
Insulin	1.00	0.96 - 1.05	0.91
sCD14	1.00	0.89 - 1.14	0.95
sCD163	1.03	0.95 - 1.12	0.46
MCP-1	1.12	1.02 - 1.23	0.02
IL-6	1.07	1.02 - 1.11	<0.01
LpPLA2	1.09	1.02 - 1.18	0.01
OxLDL	1.08	0.98 - 1.18	0.13
hsCRP	1.02	0.98 - 1.06	0.28

c) Presence of Vulnerable Plaque

	Univariate Models*		
**Biomarker#**	**OR**	**95%CI**	**p-value**
Insulin	1.00	0.95 - 1.06	0.86
sCD14	0.96	0.83 - 1.10	0.55
sCD163	0.97	0.89 - 1.06	0.57
MCP-1	1.15	1.04 - 1.27	<0.01
IL-6	1.07	1.02 - 1.12	<0.01
LpPLA2	1.03	0.95 - 1.11	0.51
OxLDL	1.15	1.03 - 1.28	0.01
hsCRP	1.05	1.01 - 1.09	0.03

© 2021 Hoffmann U et al. *JAMA Network Open.*

d) Leaman Score >5

	Univariate Models*		
**Biomarker#**	**OR**	**95%CI**	**p-value**
Insulin	1.03	0.97 - 1.10	0.31
sCD14	1.01	0.86 - 1.19	0.91
sCD163	1.07	0.97 - 1.18	0.20
MCP-1	1.10	0.98 - 1.24	0.09
IL-6	1.06	1.01 - 1.11	0.03
LPPLA2	1.11	1.01 - 1.22	0.04
OxLDL	1.04	0.92 - 1.17	0.56
hsCRP	1.07	1.03 - 1.12	<0.01

All Biomarker log2-transformed / 0.32192809, to give effects per 25% increase of biomarker value
* Each line presents one model

Abbreviations: hsCRP, high sensitivity C-reactive protein; IL-6, interleukin-6; LpPLA2, lipoprotein-associated phospholipase A2; MCP-1, monocyte chemoattractant protein-1; oxLDL, oxidized LDL; sCD14, soluble CD14; sCD163, soluble CD163.

© 2021 Hoffmann U et al. *JAMA Network Open.*

eTable 6. Multivariate Regression Modeling for Coronary Artery Disease Parameters

a) Calcium Score >0

	Multivariate Model 1*			Multivariate Model 2**			Multivariate Model 3***		
	**aOR**	**95%CI**	**p-value**	**aOR**	**95%CI**	**p-value**	**aOR**	**95%CI**	**p-value**
**Biomarker#**									
MCP-1	1.06	0.96 - 1.17	0.27	1.02	0.92 - 1.13	0.75	1.03	0.92 - 1.14	0.65
IL-6	1.06	1.01 - 1.11	0.02	1.06	1.01 - 1.12	0.01	1.07	1.02 - 1.12	<0.01
LpPLA2	1.11	1.03 - 1.20	<0.01	1.07	0.99 - 1.16	0.10	1.06	0.97 - 1.15	0.19
**Demographics/ CV Risk**									
ASCVD risk	1.14	1.08 - 1.20	<0.01	1.09	1.01 - 1.17	0.02			
Age				1.06	1.02 - 1.10	<0.01	1.10	1.06 - 1.13	<0.01
Male				1.43	0.83 - 2.47	0.20	1.96	1.19 - 3.24	<0.01
Race									
White				Base			Base		
Black				0.60	0.39 - 0.93	0.02	0.61	0.40 - 0.93	0.02
Asian				2.44	0.62 - 9.53	0.20	3.21	0.80 - 12.84	0.10
Other				0.88	0.49 - 1.57	0.66	0.92	0.51 - 1.66	0.78
LDL-C							1.00	1.00 - 1.01	0.10
Hypertension							1.57	1.10 - 2.24	0.01
Current Smoking							1.83	1.20 - 2.81	<0.01
**HIV Parameters**									
Total ART Use duration (years)									
<5	Base			Base			Base		
5-10	0.84	0.49 - 1.42	0.51	0.87	0.51 - 1.49	0.61	0.75	0.44 - 1.31	0.32
>10	0.99	0.61 - 1.60	0.97	0.97	0.59 - 1.57	0.89	0.89	0.55 - 1.46	0.66
CD4									
<350	Base			Base			Base		
350-499	1.05	0.61 - 1.81	0.87	1.05	0.60 - 1.84	0.87	1.00	0.57 - 1.77	0.99
⩾500	0.88	0.54 - 1.43	0.61	0.88	0.53 - 1.44	0.61	0.85	0.51 - 1.41	0.52
Nadir CD4									
<50	Base			Base			Base		
50-199	0.65	0.41 - 1.03	0.06	0.59	0.37 - 0.95	0.03	0.57	0.36 - 0.92	0.02
200-349	0.79	0.49 - 1.27	0.33	0.67	0.41 - 1.10	0.11	0.72	0.44 - 1.19	0.20
⩾350	0.59	0.34 - 1.02	0.06	0.56	0.32 - 0.98	0.04	0.58	0.32 - 1.02	0.06
Unknown	0.67	0.25 - 1.81	0.43	0.56	0.20 - 1.56	0.27	0.53	0.19 - 1.47	0.22

© 2021 Hoffmann U et al. *JAMA Network Open.*

b) Presence of Vulnerable Plaque

	Multivariate Model 1*			Multivariate Model 2**			Multivariate Model 3***		
	aOR	95%CI	p-value	aOR	95%CI	p-value	aOR	95%CI	p-value
**Biomarker#**									
MCP-1	1.09	0.98 - 1.21	0.12	1.06	0.95 - 1.19	0.29	1.08	0.96 - 1.21	0.20
IL-6	1.05	1.00 - 1.11	0.06	1.06	1.00 - 1.12	0.04	1.06	1.00 - 1.12	0.03
oxLDL	1.10	0.98 - 1.23	0.12	1.10	0.98 - 1.24	0.09	1.07	0.93 - 1.24	0.34
hsCRP	1.03	0.98 - 1.08	0.20	1.05	1.00 - 1.10	0.06	1.04	0.99 - 1.09	0.11
**Demographics/ CV Risk**									
ASCVD risk	1.12	1.06 - 1.18	<0.01	1.07	0.99 - 1.16	0.09			
Age				1.05	1.01 - 1.09	0.02	1.08	1.04 - 1.12	<0.01
Male				1.77	0.94 - 3.36	0.09	2.28	1.26 - 4.12	<0.01
Race									
White				Base			Base		
Black				0.76	0.49 - 1.19	0.23	0.75	0.48 - 1.16	0.19
Asian				4.25	1.07 - 16.95	0.04	5.42	1.33 - 22.16	0.02
Other				0.89	0.45 - 1.76	0.75	0.90	0.45 - 1.80	0.77
LDL-C							1.01	1.00 - 1.01	0.17
Hypertension							1.46	0.98 - 2.16	0.06
Current Smoking							1.87	1.19 - 2.96	<0.01
**HIV Parameters**									
Total ART Use duration (years)									
<5	Base			Base			Base		
5-10	0.85	0.47 - 1.52	0.58	0.84	0.46 - 1.53	0.57	0.74	0.40 - 1.35	0.32
>10	0.82	0.48 - 1.41	0.47	0.78	0.45 - 1.35	0.37	0.72	0.41 - 1.26	0.25
CD4									
<350	Base			Base			Base		
350-499	0.67	0.34 - 1.32	0.25	0.70	0.36 - 1.39	0.31	0.71	0.35 - 1.42	0.33
⩾500	1.40	0.80 - 2.46	0.24	1.42	0.80 - 2.52	0.23	1.42	0.79 - 2.54	0.24
Nadir CD4									
<50	Base			Base			Base		
50-199	0.84	0.51 - 1.40	0.51	0.80	0.48 - 1.33	0.39	0.75	0.44 - 1.26	0.28
200-349	0.74	0.43 - 1.27	0.27	0.66	0.38 - 1.15	0.14	0.68	0.39 - 1.20	0.18
⩾350	0.91	0.50 - 1.64	0.74	0.89	0.48 - 1.63	0.70	0.83	0.45 - 1.55	0.57
Unknown	0.68	0.21 - 2.22	0.52	0.59	0.17 - 1.99	0.39	0.56	0.17 - 1.88	0.35

© 2021 Hoffmann U et al. *JAMA Network Open.*

c) Leaman Score >5

	Multivariate Model 1*			Multivariate Model 2**			Multivariate Model 3***		
	aOR	95%CI	p-value	aOR	95%CI	p-value	aOR	95%CI	p-value
**Biomarker[#]**									
IL-6	1.04	0.98 - 1.11	0.18	1.04	0.98 - 1.11	0.20	1.05	0.98 - 1.11	0.16
LpPLA2	1.12	1.01 - 1.24	0.03	1.08	0.97 - 1.21	0.16	1.08	0.96 - 1.21	0.21
hsCRP	1.07	1.01 - 1.12	0.01	1.08	1.03 - 1.14	<0.01	1.08	1.02 - 1.14	<0.01
**Demographics/ CV Risk**									
ASCVD risk	1.14	1.07 - 1.21	<0.01	1.08	0.99 - 1.18	0.07			
Age				1.07	1.02 - 1.11	<0.01	1.11	1.06 - 1.15	<0.01
Male				1.78	0.83 - 3.82	0.14	2.43	1.19 - 4.96	0.02
Race									
White				Base			Base		
Black				0.67	0.39 - 1.15	0.15	0.62	0.36 - 1.06	0.08
Asian				6.11	1.39 - 26.86	0.02	8.38	1.84 - 38.29	<0.01
Other				1.02	0.47 - 2.19	0.97	0.99	0.45 - 2.16	0.98
LDL-C							1.01	1.00 - 1.01	0.19
Hypertension							1.44	0.91 - 2.26	0.12
Current Smoking							2.33	1.38 - 3.92	<0.01
**HIV Parameters**									
Total ART Use duration (years)									
<5	Base			Base			Base		
5-10	0.51	0.25 - 1.01	0.06	0.50	0.25 - 1.02	0.06	0.44	0.21 - 0.91	0.03
>10	0.86	0.48 - 1.55	0.62	0.82	0.45 - 1.50	0.53	0.75	0.41 - 1.38	0.35
CD4									
<350	Base			Base			Base		
350-499	0.52	0.25 - 1.10	0.09	0.53	0.25 - 1.12	0.10	0.48	0.23 - 1.03	0.06
⩾500	0.77	0.43 - 1.40	0.40	0.78	0.42 - 1.43	0.42	0.71	0.38 - 1.31	0.27
Nadir CD4									
<50	Base			Base			Base		
50-199	0.64	0.36 - 1.14	0.13	0.58	0.32 - 1.05	0.07	0.51	0.28 - 0.93	0.03
200-349	0.67	0.36 - 1.24	0.20	0.59	0.31 - 1.11	0.10	0.59	0.31 - 1.10	0.10
⩾350	0.96	0.50 - 1.87	0.92	0.95	0.48 - 1.86	0.87	0.83	0.42 - 1.65	0.60
Unknown	0.68	0.18 - 2.54	0.56	0.51	0.13 - 2.06	0.35	0.48	0.12 - 1.91	0.30

All Biomarker log-transformed using Log2 / 0.32192809, to give effects per 25% increase of biomarker value
* All Biomarker that were significant in univariate analysis, ASCVD risk, HIV Parameters (ART duration, CD4, Nadir CD4)
** Same variables as Model 1 plus age, male, race
*** Same variables as Model 2 minus ASCVD and plus LDL, hypertension, current smoking
Abbreviations: ART, antiretroviral therapy; ASCVD, atherosclerotic cardiovascular disease; hsCRP, high sensitivity C-reactive protein; IL-6, interleukin-6; LpPLA2, lipoprotein-associated phospholipase A2; MCP-1, monocyte chemoattractant protein-1; oxLDL, oxidized LDL.

© 2021 Hoffmann U et al. *JAMA Network Open.*

eTable 7. Multivariate Regression Modeling for Coronary Artery Disease Parameters Including All Biomarkers and Covariates

a) Presence of Plaque

	Multivariate Model 1*			Multivariate Model 2**			Multivariate Model 3***		
	aOR	95%CI	p-value	aOR	95%CI	p-value	aOR	95%CI	p-value
**Biomarker#**									
Insulin	1.01	0.96 - 1.06	0.74	1.01	0.96 - 1.06	0.72	1.03	0.98 - 1.09	0.23
sCD14	0.93	0.82 - 1.06	0.29	0.94	0.82 - 1.08	0.40	0.95	0.83 - 1.09	0.44
sCD163	1.02	0.94 - 1.11	0.69	1.01	0.93 - 1.10	0.81	1.01	0.92 - 1.10	0.90
MCP-1	1.10	1.00 - 1.20	0.06	1.06	0.96 - 1.17	0.29	1.07	0.97 - 1.19	0.19
IL-6	1.05	1.00 - 1.10	0.08	1.05	1.00 - 1.11	0.07	1.06	1.00 - 1.12	0.04
LpPLA2	1.18	1.09 - 1.27	<0.01	1.13	1.04 - 1.22	<0.01	1.10	1.02 - 1.20	0.02
oxLDL	1.05	0.95 - 1.17	0.30	1.07	0.96 - 1.19	0.20	0.98	0.87 - 1.12	0.81
hsCRP	1.02	0.98 - 1.07	0.33	1.04	0.99 - 1.08	0.11	1.03	0.98 - 1.07	0.24
**Demographics/ CV Risk**									
ASCVD risk	1.15	1.09 - 1.22	<0.01	1.08	1.00 - 1.16	0.04			
Age				1.07	1.03 - 1.11	<0.01	1.10	1.07 - 1.14	<0.01
Male				1.82	1.08 - 3.07	0.03	2.49	1.54 - 4.05	<0.01
Race									
White				Base			Base		
Black				0.64	0.42 - 0.98	0.04	0.64	0.43 - 0.96	0.03
Asian				1.45	0.36 - 5.77	0.60	1.60	0.39 - 6.54	0.52
Other				0.99	0.57 - 1.71	0.96	1.02	0.58 - 1.80	0.94
LDL-C							1.01	1.00 - 1.02	<0.01
Hypertension							1.52	1.07 - 2.16	0.02
Current Smoking							1.75	1.16 - 2.64	<0.01
**HIV Parameters**									
Total ART Use duration (years)									
<5	Base			Base			Base		
5-10	0.86	0.52 - 1.41	0.55	0.90	0.54 - 1.51	0.70	0.80	0.47 - 1.34	0.40
>10	1.04	0.66 - 1.65	0.85	0.99	0.62 - 1.57	0.95	0.92	0.57 - 1.47	0.72
CD4									
<350	Base			Base			Base		
350-499	0.83	0.48 - 1.42	0.49	0.84	0.48 - 1.45	0.53	0.83	0.47 - 1.45	0.51
⩾500	0.98	0.61 - 1.59	0.94	1.00	0.61 - 1.63	1.00	0.98	0.59 - 1.62	0.93
Nadir CD4									
<50	Base			Base			Base		
50-199	0.63	0.40 - 0.98	0.04	0.58	0.37 - 0.91	0.02	0.55	0.34 - 0.87	0.01
200-349	0.73	0.46 - 1.17	0.19	0.65	0.40 - 1.04	0.08	0.69	0.42 - 1.12	0.13
⩾350	0.70	0.41 - 1.18	0.18	0.67	0.39 - 1.16	0.15	0.67	0.39 - 1.17	0.16

© 2021 Hoffmann U et al. *JAMA Network Open.*

Unknown	0.54	0.21 - 1.40	0.21	0.44	0.16 - 1.19	0.11	0.41	0.15 - 1.13	0.08

b) Calcium Score >0

	**Multivariate Model 1***			**Multivariate Model 2****			**Multivariate Model 3*****		
	**aOR**	**95%CI**	**p-value**	**aOR**	**95%CI**	**p-value**	**aOR**	**95%CI**	**p-value**
**Biomarker**[#]									
Insulin	0.99	0.94 - 1.04	0.62	0.98	0.93 - 1.04	0.53	1.00	0.95 - 1.06	0.94
sCD14	1.05	0.91 - 1.20	0.52	1.06	0.92 - 1.22	0.44	1.06	0.92 - 1.23	0.40
sCD163	1.02	0.93 - 1.11	0.70	1.01	0.93 - 1.11	0.77	1.01	0.92 - 1.11	0.87
MCP-1	1.05	0.95 - 1.17	0.33	1.01	0.91 - 1.12	0.88	1.02	0.92 - 1.14	0.69
IL-6	1.06	1.01 - 1.11	0.02	1.06	1.01 - 1.12	0.02	1.07	1.02 - 1.13	0.01
LpPLA2	1.11	1.02 - 1.20	0.01	1.06	0.98 - 1.16	0.16	1.05	0.96 - 1.14	0.28
oxLDL	1.01	0.91 - 1.12	0.84	1.02	0.92 - 1.14	0.70	0.97	0.85 - 1.11	0.67
hsCRP	1.00	0.96 - 1.05	0.96	1.01	0.97 - 1.06	0.56	1.00	0.96 - 1.05	0.94
**Demographics/ CV Risk**									
ASCVD risk	1.14	1.08 - 1.20	<0.01	1.09	1.01 - 1.18	0.02			
Age				1.06	1.02 - 1.10	<0.01	1.10	1.06 - 1.13	<0.01
Male				1.49	0.85 - 2.61	0.17	2.09	1.24 - 3.51	<0.01
Race									
White				Base			Base		
Black				0.59	0.38 - 0.92	0.02	0.61	0.39 - 0.93	0.02
Asian				2.67	0.67 - 10.58	0.16	3.21	0.79 - 13.07	0.10
Other				0.89	0.50 - 1.60	0.69	0.92	0.51 - 1.68	0.79
LDL-C							1.01	1.00 - 1.01	0.08
Hypertension							1.61	1.12 - 2.32	0.01
Current Smoking							1.81	1.18 - 2.80	<0.01
**HIV Parameters**									
Total ART Use duration (years)									
<5	Base			Base			Base		
5-10	0.85	0.50 - 1.44	0.54	0.88	0.51 - 1.52	0.65	0.76	0.44 - 1.33	0.34
>10	0.99	0.61 - 1.60	0.97	0.96	0.59 - 1.57	0.88	0.89	0.54 - 1.46	0.64
CD4									
<350	Base			Base			Base		
350-499	1.04	0.60 - 1.81	0.88	1.06	0.60 - 1.86	0.85	1.03	0.58 - 1.83	0.92
⩾500	0.88	0.54 - 1.44	0.62	0.88	0.53 - 1.45	0.61	0.86	0.52 - 1.43	0.56
Nadir CD4									
<50	Base			Base			Base		
50-199	0.65	0.41 - 1.03	0.07	0.60	0.37 - 0.95	0.03	0.57	0.35 - 0.92	0.02
200-349	0.79	0.49 - 1.28	0.34	0.68	0.42 - 1.11	0.12	0.73	0.44 - 1.20	0.21
⩾350	0.59	0.34 - 1.04	0.07	0.57	0.32 - 1.01	0.05	0.58	0.32 - 1.03	0.06
Unknown	0.67	0.25 - 1.81	0.44	0.56	0.20 - 1.56	0.27	0.52	0.19 - 1.45	0.21

© 2021 Hoffmann U et al. *JAMA Network Open.*

c) Presence of Vulnerable Plaque

	Multivariate Model 1*			Multivariate Model 2**			Multivariate Model 3***		
	**aOR**	**95%CI**	**p-value**	**aOR**	**95%CI**	**p-value**	**aOR**	**95%CI**	**p-value**
**Biomarker#**									
Insulin	0.99	0.93 - 1.05	0.71	0.99	0.93 - 1.05	0.63	1.01	0.95 - 1.07	0.85
sCD14	1.01	0.86 - 1.18	0.94	1.01	0.87 - 1.19	0.86	1.00	0.85 - 1.18	0.96
sCD163	0.98	0.89 - 1.08	0.74	0.99	0.89 - 1.09	0.82	0.98	0.88 - 1.08	0.64
MCP-1	1.09	0.98 - 1.22	0.11	1.07	0.95 - 1.19	0.28	1.08	0.96 - 1.22	0.18
IL-6	1.05	1.00 - 1.11	0.06	1.06	1.00 - 1.11	0.05	1.06	1.00 - 1.12	0.04
LpPLA2	1.01	0.93 - 1.09	0.86	0.97	0.89 - 1.07	0.57	0.97	0.88 - 1.06	0.47
oxLDL	1.09	0.97 - 1.23	0.14	1.11	0.99 - 1.26	0.08	1.07	0.92 - 1.24	0.39
hsCRP	1.03	0.98 - 1.08	0.20	1.05	1.00 - 1.10	0.07	1.04	0.99 - 1.09	0.11
**Demographics/ CV Risk**									
ASCVD risk	1.12	1.06 - 1.19	<0.01	1.07	0.99 - 1.16	0.08			
Age				1.05	1.01 - 1.09	0.02	1.08	1.04 - 1.12	<0.01
Male				1.76	0.92 - 3.35	0.09	2.31	1.26 - 4.25	<0.01
Race									
White				Base			Base		
Black				0.71	0.43 - 1.15	0.17	0.70	0.44 - 1.12	0.13
Asian				4.10	1.01 - 16.67	0.05	4.98	1.20 - 20.71	0.03
Other				0.89	0.45 - 1.76	0.74	0.90	0.45 - 1.79	0.76
LDL-C							1.01	1.00 - 1.01	0.13
Hypertension							1.47	0.99 - 2.18	0.06
Current Smoking							1.90	1.19 - 3.02	<0.01
**HIV Parameters**									
Total ART Use duration (years)									
<5	Base			Base			Base		
5-10	0.84	0.46 - 1.51	0.56	0.84	0.46 - 1.52	0.56	0.72	0.39 - 1.33	0.30
>10	0.82	0.47 - 1.41	0.47	0.78	0.45 - 1.35	0.37	0.71	0.40 - 1.24	0.23
CD4									
<350	Base			Base			Base		
350-499	0.67	0.34 - 1.33	0.25	0.69	0.35 - 1.37	0.29	0.70	0.35 - 1.41	0.32
⩾500	1.40	0.80 - 2.45	0.24	1.42	0.80 - 2.51	0.23	1.42	0.79 - 2.54	0.24
Nadir CD4									
<50	Base			Base			Base		
50-199	0.84	0.50 - 1.39	0.49	0.79	0.47 - 1.33	0.39	0.75	0.44 - 1.27	0.28
200-349	0.73	0.42 - 1.26	0.26	0.66	0.38 - 1.15	0.14	0.68	0.39 - 1.20	0.19
⩾350	0.90	0.50 - 1.64	0.73	0.89	0.48 - 1.63	0.70	0.84	0.45 - 1.55	0.57

© 2021 Hoffmann U et al. *JAMA Network Open.*

Unknown	0.68	0.21 - 2.23	0.52	0.59	0.17 - 2.00	0.40	0.56	0.17 - 1.89	0.35

d) Leaman Score >5

	**Multivariate Model 1***			**Multivariate Model 2****			**Multivariate Model 3*****		
	**aOR**	**95%CI**	**p-value**	**aOR**	**95%CI**	**p-value**	**aOR**	**95%CI**	**p-value**
**Biomarker#**									
Insulin	1.01	0.95 - 1.08	0.73	1.01	0.94 - 1.08	0.84	1.04	0.97 - 1.11	0.28
sCD14	1.01	0.84 - 1.20	0.95	1.01	0.84 - 1.21	0.94	0.99	0.82 - 1.20	0.92
sCD163	1.05	0.94 - 1.18	0.37	1.07	0.95 - 1.20	0.28	1.05	0.93 - 1.18	0.42
MCP-1	1.04	0.91 - 1.18	0.58	1.00	0.87 - 1.15	0.99	1.01	0.87 - 1.15	0.94
IL-6	1.04	0.97 - 1.10	0.29	1.04	0.97 - 1.11	0.24	1.04	0.98 - 1.11	0.20
LpPLA2	1.12	1.00 - 1.24	0.04	1.08	0.97 - 1.21	0.17	1.07	0.96 - 1.20	0.24
oxLDL	0.95	0.83 - 1.09	0.46	0.97	0.84 - 1.12	0.65	0.90	0.75 - 1.07	0.24
hsCRP	1.07	1.01 - 1.12	0.02	1.08	1.03 - 1.14	<0.01	1.08	1.02 - 1.14	<0.01
**Demographics/ CV Risk**									
ASCVD risk	1.14	1.07 - 1.22	<0.01	1.09	0.99 - 1.19	0.07			
Age				1.06	1.02 - 1.11	<0.01	1.11	1.07 - 1.16	<0.01
Male				1.88	0.86 - 4.10	0.11	2.72	1.30 - 5.69	<0.01
Race									
White				Base			Base		
Black				0.69	0.39 - 1.21	0.19	0.64	0.37 - 1.12	0.12
Asian				6.52	1.46 - 29.07	0.01	8.22	1.78 - 38.04	<0.01
Other				0.97	0.45 - 2.10	0.93	0.94	0.43 - 2.07	0.88
LDL-C							1.01	1.00 - 1.02	0.07
Hypertension							1.44	0.91 - 2.29	0.12
Current Smoking							2.46	1.43 - 4.23	<0.01
**HIV Parameters**									
Total ART Use duration (years)									
<5	Base			Base			Base		
5-10	0.51*	0.25 - 1.02	0.06	0.51	0.25 - 1.05	0.07	0.44	0.21 - 0.91	0.03
>10	0.87	0.48 - 1.57	0.64	0.84	0.46 - 1.54	0.58	0.74	0.40 - 1.38	0.35
CD4									
<350	Base			Base			Base		
350-499	0.53*	0.25 - 1.11	0.09	0.52	0.24 - 1.12	0.10	0.50	0.23 - 1.07	0.08
⩾500	0.81	0.44 - 1.48	0.49	0.80	0.43 - 1.48	0.47	0.74	0.40 - 1.39	0.36
Nadir CD4									
<50	Base			Base			Base		
50-199	0.64	0.36 - 1.14	0.13	0.59	0.32 - 1.06	0.08	0.52	0.29 - 0.95	0.03
200-349	0.66	0.36 - 1.23	0.19	0.58	0.31 - 1.10	0.09	0.59	0.31 - 1.11	0.10

© 2021 Hoffmann U et al. *JAMA Network Open.*

⩾350	0.94	0.48 - 1.84	0.86	0.94	0.47 - 1.85	0.85	0.81	0.41 - 1.63	0.56
Unknown	0.64	0.17 - 2.41	0.51	0.50	0.12 - 2.00	0.32	0.45	0.11 - 1.84	0.27

All Biomarker log-transformed using Log2 / 0.32192809, to give effects per 25% increase of biomarker value
* All Biomarker, ASCVD risk, HIV Parameters (ART duration, CD4, Nadir CD4)
** Same variables as Model 1 plus age, male, race
*** Same variables as Model 2 minus ASCVD and plus LDL, hypertension, current smoking
Abbreviations: ART, antiretroviral therapy; ASCVD, atherosclerotic cardiovascular disease; hsCRP, high sensitivity C-reactive protein; IL-6, interleukin-6; LpPLA2, lipoprotein-associated phospholipase A2; MCP-1, monocyte chemoattractant protein-1; oxLDL, oxidized LDL.

© 2021 Hoffmann U et al. *JAMA Network Open.*

eTable 8. Multivariate Regression Modeling for Coronary Artery Disease Parameters per SD of Log Transformed Values

a) Presence of Plaque

	Multivariate Model 1*			Multivariate Model 2**			Multivariate Model 3***		
	**aOR**	**95%CI**	**p-value**	**aOR**	**95%CI**	**p-value**	**aOR**	**95%CI**	**p-value**
**Biomarker#**									
MCP-1	1.18	1.00 - 1.38	0.05	1.10	0.93 - 1.30	0.25	1.13	0.96 - 1.35	0.15
IL-6	1.20	1.02 - 1.41	0.03	1.23	1.04 - 1.46	0.01	1.25	1.06 - 1.48	0.01
LpPLA2	1.45	1.22 - 1.71	<0.01	1.31	1.09 - 1.57	<0.01	1.26	1.05 - 1.52	0.01
oxLDL	1.12	0.95 - 1.31	0.18	1.15	0.97 - 1.35	0.10	1.02	0.84 - 1.25	0.82
**Demographics/ CV Risk**									
ASCVD risk	1.16	1.10 - 1.22	<0.01	1.09	1.01 - 1.17	0.02			
Age				1.07	1.03 - 1.11	<0.01	1.10	1.07 - 1.14	<0.01
Male				1.70	1.02 - 2.83	0.04	2.37	1.49 - 3.78	<0.01
Race									
White				Base			Base		
Black				0.63	0.42 - 0.96	0.03	0.64	0.43 - 0.96	0.03
Asian				1.39	0.35 - 5.51	0.64	1.63	0.40 - 6.65	0.50
Other				0.98	0.57 - 1.70	0.95	1.04	0.59 - 1.82	0.89
LDL							1.01	1.00 - 1.02	0.02
Hypertension							1.54	1.08 - 2.18	0.02
Current Smoking							1.70	1.13 - 2.54	0.01
**HIV Parameters**									
Total ART Use duration (years)									
<5	Base			Base			Base		
5-10	0.84	0.51 - 1.37	0.48	0.88	0.53 - 1.46	0.61	0.78	0.47 - 1.31	0.36
>10	1.03	0.65 - 1.62	0.90	0.98	0.62 - 1.55	0.93	0.92	0.58 - 1.47	0.74
CD4									
<350	Base			Base			Base		
350-499	0.83	0.49 - 1.42	0.50	0.83	0.48 - 1.44	0.51	0.82	0.47 - 1.43	0.48
≥500	0.97	0.60 - 1.57	0.91	0.99	0.60 - 1.61	0.96	0.97	0.59 - 1.59	0.89
Nadir CD4									
<50	Base			Base			Base		
50-199	0.62	0.40 - 0.96	0.03	0.57	0.36 - 0.89	0.01	0.54	0.34 - 0.85	<0.01
200-349	0.72	0.45 - 1.14	0.16	0.63	0.39 - 1.01	0.06	0.66	0.40 - 1.07	0.09
≥350	0.69	0.40 - 1.16	0.16	0.66	0.38 - 1.12	0.13	0.66	0.38 - 1.15	0.14
Unknown	0.54	0.21 - 1.40	0.20	0.45	0.17 - 1.20	0.11	0.42	0.15 - 1.13	0.09

© 2021 Hoffmann U et al. *JAMA Network Open.*

b) Calcium Score >0

	Multivariate Model 1*			Multivariate Model 2**			Multivariate Model 3***		
	**aOR**	**95%CI**	**p-value**	**aOR**	**95%CI**	**p-value**	**aOR**	**95%CI**	**p-value**
**Biomarker#**									
MCP-1	1.10	0.93 - 1.31	0.26	1.03	0.86 - 1.23	0.75	1.04	0.87 - 1.26	0.65
IL-6	1.21	1.03 - 1.42	0.02	1.23	1.05 - 1.45	0.01	1.25	1.06 - 1.47	<0.01
LpPLA2	1.27	1.07 - 1.51	<0.01	1.17	0.97 - 1.41	0.10	1.14	0.94 - 1.38	0.19
**Demographics/ CV Risk**									
ASCVD risk	1.14	1.08 - 1.20	<0.01	1.09	1.01 - 1.17	0.02			
Age				1.06	1.02 - 1.10	<0.01	1.10	1.06 - 1.13	<0.01
Male				1.43	0.83 - 2.47	0.20	1.96	1.19 - 3.24	<0.01
Race									
White				Base			Base		
Black				0.60	0.39 - 0.93	0.02	0.61	0.40 - 0.93	0.02
Asian				2.44	0.62 - 9.53	0.20	3.21	0.80 - 12.84	0.10
Other				0.88	0.49 - 1.57	0.66	0.92	0.51 - 1.66	0.78
LDL							1.00	1.00 - 1.01	0.10
Hypertension							1.57	1.10 - 2.24	0.01
Current Smoking							1.83	1.20 - 2.81	<0.01
**HIV Parameters**									
Total ART Use duration (years)									
<5	Base			Base			Base		
5-10	0.84	0.49 - 1.42	0.51	0.87	0.51 - 1.49	0.61	0.75	0.44 - 1.31	0.32
>10	0.99	0.61 - 1.60	0.97	0.97	0.59 - 1.57	0.89	0.89	0.55 - 1.46	0.66
CD4									
<350	Base			Base			Base		
350-499	1.05	0.61 - 1.81	0.87	1.05	0.60 - 1.84	0.87	1.00	0.57 - 1.77	0.99
≥500	0.88	0.54 - 1.43	0.61	0.88	0.53 - 1.44	0.61	0.85	0.51 - 1.41	0.52
Nadir CD4									
<50	Base			Base			Base		
50-199	0.65	0.41 - 1.03	0.06	0.59	0.37 - 0.95	0.03	0.57	0.36 - 0.92	0.02
200-349	0.79	0.49 - 1.27	0.33	0.67	0.41 - 1.10	0.11	0.72	0.44 - 1.19	0.20
≥350	0.59	0.34 - 1.02	0.06	0.56	0.32 - 0.98	0.04	0.58	0.32 - 1.02	0.06
Unknown	0.67	0.25 - 1.81	0.43	0.56	0.20 - 1.56	0.27	0.53	0.19 - 1.47	0.22

© 2021 Hoffmann U et al. *JAMA Network Open.*

c) Presence of Vulnerable Plaque

	Multivariate Model 1*			Multivariate Model 2**			Multivariate Model 3***		
	aOR	95%CI	p-value	aOR	95%CI	p-value	aOR	95%CI	p-value
**Biomarker#**									
MCP-1	1.16	0.96 - 1.39	0.12	1.11	0.92 - 1.35	0.29	1.14	0.94 - 1.39	0.20
IL-6	1.18	0.99 - 1.41	0.06	1.20	1.00 - 1.44	0.04	1.22	1.02 - 1.46	0.03
oxLDL	1.16	0.96 - 1.40	0.12	1.18	0.97 - 1.43	0.09	1.12	0.88 - 1.43	0.34
hsCRP	1.13	0.94 - 1.34	0.20	1.19	0.99 - 1.42	0.06	1.16	0.97 - 1.40	0.11
**Demographics/ CV Risk**									
ASCVD risk	1.12	1.06 - 1.18	<0.01	1.07	0.99 - 1.16	0.09			
Age				1.05	1.01 - 1.09	0.02	1.08	1.04 - 1.12	<0.01
Male				1.77	0.94 - 3.36	0.08	2.28	1.26 - 4.12	<0.01
Race									
White				Base			Base		
Black				0.76	0.49 - 1.19	0.23	0.75	0.48 - 1.16	0.19
Asian				4.25	1.07 - 16.95	0.04	5.42	1.33 - 22.16	0.02
Other				0.89	0.45 - 1.76	0.75	0.90	0.45 - 1.80	0.77
LDL-C							1.01	1.00 - 1.01	0.17
Hypertension							1.46	0.98 - 2.16	0.06
Current Smoking							1.87	1.19 - 2.96	<0.01
**HIV Parameters**									
Total ART Use duration (years)									
<5	Base			Base			Base		
5-10	0.85	0.47 - 1.52	0.58	0.84	0.46 - 1.53	0.57	0.74	0.40 - 1.35	0.32
>10	0.82	0.48 - 1.41	0.47	0.78	0.45 - 1.35	0.37	0.72	0.41 - 1.26	0.25
CD4									
<350	Base			Base			Base		
350-499	0.67	0.34 - 1.32	0.25	0.70	0.36 - 1.39	0.31	0.71	0.35 - 1.42	0.33
≥500	1.40	0.80 - 2.46	0.24	1.42	0.80 - 2.52	0.23	1.42	0.79 - 2.54	0.24
Nadir CD4									
<50	Base			Base			Base		
50-199	0.84	0.51 - 1.40	0.51	0.80	0.48 - 1.33	0.39	0.75	0.44 - 1.26	0.28
200-349	0.74	0.43 - 1.27	0.27	0.66	0.38 - 1.15	0.14	0.68	0.39 - 1.20	0.18
≥350	0.91	0.50 - 1.64	0.74	0.89	0.48 - 1.63	0.70	0.83	0.45 - 1.55	0.57
Unknown	0.68	0.21 - 2.22	0.52	0.59	0.17 - 1.99	0.39	0.56	0.17 - 1.88	0.35

© 2021 Hoffmann U et al. *JAMA Network Open.*

d) Leaman Score >5

	Multivariate Model 1*			Multivariate Model 2**			Multivariate Model 3***		
	aOR	95%CI	p-value	aOR	95%CI	p-value	aOR	95%CI	p-value
**Biomarker#**									
IL-6	1.15	0.94 - 1.41	0.18	1.15	0.93 - 1.41	0.20	1.16	0.94 - 1.43	0.16
LpPLA2	1.29	1.02 - 1.63	0.03	1.20	0.93 - 1.53	0.16	1.18	0.91 - 1.54	0.21
hsCRP	1.27	1.05 - 1.54	0.01	1.36	1.12 - 1.66	<0.01	1.34	1.09 - 1.63	<0.01
**Demographics/ CV Risk**									
ASCVD risk	1.14	1.07 - 1.21	<0.01	1.08	0.99 - 1.18	0.07			
Age				1.07	1.02 - 1.11	<0.01	1.11	1.06 - 1.15	<0.01
Male				1.78	0.83 - 3.82	0.14	2.43	1.19 - 4.96	0.02
Race									
White				Base			Base		
Black				0.67	0.39 - 1.15	0.15	0.62	0.36 - 1.06	0.08
Asian				6.11	1.39 - 26.86	0.02	8.38	1.84 - 38.29	<0.01
Other				1.02	0.47 - 2.19	0.97	0.99	0.45 - 2.16	0.98
LDL-C							1.01	1.00 - 1.01	0.19
Hypertension							1.44	0.91 - 2.26	0.12
Current Smoking							2.33	1.38 - 3.92	<0.01
**HIV Parameters**									
Total ART Use duration (years)									
<5	Base			Base			Base		
5-10	0.51	0.25 - 1.01	0.06	0.50	0.25 - 1.02	0.06	0.44	0.21 - 0.91	0.03
>10	0.86	0.48 - 1.55	0.62	0.82	0.45 - 1.50	0.53	0.75	0.41 - 1.38	0.35
CD4									
<350	Base			Base			Base		
350-499	0.52	0.25 - 1.10	0.09	0.53	0.25 - 1.12	0.10	0.48	0.23 - 1.03	0.06
≥500	0.77	0.43 - 1.40	0.40	0.78	0.42 - 1.43	0.42	0.71	0.38 - 1.31	0.27
Nadir CD4									
<50	Base			Base			Base		
50-199	0.64	0.36 - 1.14	0.13	0.58	0.32 - 1.05	0.07	0.51	0.28 - 0.93	0.03
200-349	0.67	0.36 - 1.24	0.20	0.59	0.31 - 1.11	0.10	0.59	0.31 - 1.10	0.10
≥350	0.96	0.50 - 1.87	0.92	0.95	0.48 - 1.86	0.87	0.83	0.42 - 1.65	0.60
Unknown	0.68	0.18 - 2.54	0.56	0.51	0.13 - 2.06	0.35	0.48	0.12 - 1.91	0.30

All Biomarker log-transformed and standardized, to give effects per SD of the log-transformed values

* All Biomarker that were significant in univariate analysis, ASCVD risk, HIV parameters (ART duration, CD4, Nadir CD4)

** Same variables as Model 1 plus age, male, race

*** Same variables as Model 2 minus ASCVD and plus LDL, hypertension, current smoking

Abbreviations: ART, antiretroviral therapy; ASCVD, atherosclerotic cardiovascular disease; hsCRP, high sensitivity C-reactive protein; IL-6, interleukin-6; LpPLA2, lipoprotein-associated phospholipase A2; MCP-1, monocyte chemoattractant protein-1; oxLDL, oxidized LDL.

© 2021 Hoffmann U et al. *JAMA Network Open.*
