# Supplementary material for: Assessment of Coronary Artery Disease With Computed Tomography Angiography and Inflammatory and Immune Activation Biomarkers Among Adults With HIV Eligible for Primary Cardiovascular Prevention
Source: JAMA Netw Open. 2021 Jun 29;4(6):e2114923. doi: 10.1001/jamanetworkopen.2021.14923 (PMC8243232; doi:10.1001/jamanetworkopen.2021.14923)
Supplement: Supplement 2. — Nonauthor Collaborators [file jamanetwopen-e2114923-s002.pdf]

Supplemental Online Content: Nonauthor Collaborators

*Indicates required information. Only first name, last name, and suffix will appear in PubMed.

*Group Name(s): REPRIEVE trial							
***First Name and Middle Initial(s)**	***Last Name**	***Suffix (eg, Jr, III)**	Academic Degrees	Institution	Location (city, state/province, country)	Role or Contribution, eg, chair, principal investigator	Group (if more than 1 Group listed in the byline) and/or Subgroup (eg, Steering Committee)
Aleen	Khodabakhshian		MD	UCLA Care Center	Los Angeles, CA, USA	Site investigator	
Amy	Sbrolla		RN	Massachusetts General Hospital	Boston, MA, USA	Research coordinator	
Beverly E	Sha		MD	Rush University	Chicago, IL, USA	Site investigator	
Christie Lyn	Costanza		MPH, BSN, RN, CCRC	Rutgers, New Jersey Medical School	Newark, NJ, USA	Research coordinator	
Claudia A	Hawkins		MD, MPH	Northwestern University	Chicago, IL, USA	Site investigator	
Connor	Reynolds		BA	Massachusetts General Hospital	Boston, MA, USA	Research coordinator	
Cornelius N	Van Dam		MD	University of North Carolina	Greensboro, NC, USA	Site investigator	
Dan	Berrner			UCSF	San Francisco, CA, USA	Research coordinator	
David	Choi			UCLA	Los Angeles, CA, USA	Research coordinator	
Jamie L	Nemeth		RMAs	University of Pittsburgh	Pittsburgh, PA, USA	Research coordinator	
Jeffrey M	Jacobson		MD	Case Western Reserve University	Cleveland, OH, USA	Site investigator	
Joan	Gottesman		RN, BSN	Vanderbilt University Medical Center	Nashville, TN, USA	Research coordinator	
John	Dwyer		RN	UCSF	San Francisco, CA, USA	Research coordinator	
John R	Koethe		MD, MSCI	Vanderbilt University Medical Center	Nashville, TN, USA	Site investigator	
Jorge L	Santana		MD, FIDSA	University of Puerto Rico, School of Medicine	San Juan, PR, USA	Research coordinator	

*Indicates required information. Only first name, last name, and suffix will appear in PubMed.

*First Name and Middle Initial(s)	*Last Name	*Suffix (eg, Jr, III)	Academic Degrees	Institution	Location (city, state/province, country)	Role or Contribution, eg, chair, principal investigator	Group (if more than 1 Group listed in the byline) and/or Subgroup (eg, Steering Committee)
Julie	Pasternak		MBA, BSN, RN	Case Western Reserve University	Cleveland, OH, USA	Research coordinator	
Ken S	Ho		MD, MPH	University of Pittsburgh	Pittsburgh, PA, USA	Site investigator	
Magdalena E	Sobieszczyk		MD, MPH	Columbia University	New York, NY, USA	Site investigator	
Mark	Mall		RN	Rush University	Chicago, IL, USA	Research coordinator	
Moises S	Huaman		MD, MSCI	University of Cincinnati	Cincinnati, OH, USA	Site investigator	
Quynh	Truong		MD	Weill Cornell University	New York, NY, USA	Site investigator	
Rebecca	Fry		FNP	Weill Cornell University	New York, NY, USA	Research coordinator	
Robert T	O'Donnell		MD	University of Cincinnati	Cincinnati, OH, USA	Site investigator	
Roberto C	Arduino		MD	The University of Texas Health Science Center at Houston, McGovern Medical School	Houston, TX, USA	Site investigator	
Romina	Chinchay Collahua		MBA, MSc	The University of Texas Health Science Center at Houston, McGovern Medical School	Houston, TX, USA	Research coordinator	
Shaun	Barcavage		FNP	Cornell University	New York, NY, USA	Research coordinator	
Shobha	Swaminathan		MD	Rutgers, New Jersey Medical School	Newark, NJ, USA	Site investigator	
Sigrid	Perez-Frontera		MD	University of Puerto Rico, School of Medicine	San Juan, PR, USA	Site investigator	
Todd	Stroberg		RN	Cornell University	New York, NY, USA	Research coordinator	
